# Implementing a method for engineering multivalency to substantially enhance binding of clinical trial anti-SARS-CoV-2 antibodies to wildtype spike and variants of concern proteins

**DOI:** 10.1038/s41598-021-89887-w

**Published:** 2021-05-18

**Authors:** Adam Leach, Ami Miller, Emma Bentley, Giada Mattiuzzo, Jemima Thomas, Craig McAndrew, Rob Van Montfort, Terence Rabbitts

**Affiliations:** 1 Institute of Cancer Research, 15 Cotswold Road, Sutton, London, SM2 5NG UK; 2 National Institute for Biological Standards and Control, Blanche Lane, South Mimms, Hertfordshire, EN6 3QG UK; 3 Present Address: Evotec, 114 Innovation Dr, Milton Park, Abingdon, OX14 4RZ UK

**Keywords:** Biotechnology, Drug discovery, Immunology, Molecular medicine

## Abstract

Infection by the severe acute respiratory syndrome coronavirus-2 (SARS-CoV-2) causes COVID-19 disease. Therapeutic antibodies are being developed that interact with the viral spike proteins to limit viral infection of epithelium. We have applied a method to dramatically improve the performance of anti-SARS-CoV-2 antibodies by enhancing avidity through multimerization using simple engineering to yield tetrameric antibodies. We have re-engineered six anti-SARS-CoV-2 antibodies using the human p53 tetramerization domain, including three clinical trials antibodies casirivimab, imdevimab and etesevimab. The method yields tetrameric antibodies, termed quads, that retain efficient binding to the SARS-CoV-2 spike protein, show up to two orders of magnitude enhancement in neutralization of pseudovirus infection and retain potent interaction with virus variant of concern spike proteins. The tetramerization method is simple, general and its application is a powerful methodological development for SARS-CoV-2 antibodies that are currently in pre-clinical and clinical investigation.

## Introduction

There is a continued need for new antibodies for use in clinical situations such as acquired disease and infections. This requirement highlights the associated costs of both individual antibody development and production of sufficient antibody for each efficacious dose. In theory, higher potency antibodies require lower dose which reflects on the cost of goods for patient implementation. We recently described a method for increasing functional affinity through multivalent antibody engineering^1^ to significantly improve potency by avidity effects. We have now applied this methodology to enhancing anti-SARS-CoV-2 antibodies including two that are in clinical use for COVID-19.

The advent of new viruses that can infect humans is a continuing threat to the population. The emergence of SARS-CoV causing severe acute respiratory syndrome occurred in 2002 and followed in late 2019 by a novel coronavirus SARS-CoV-2 are examples^2^. Methodologies to produce the best antibodies are a critical need for keeping ahead of new, emerging infectious agents. Controlling the SARS-CoV-2 pandemic requires development of rapid, sensitive testing to determine transmission but also effective therapeutic antibodies to prevent viral entry into cells that causes the subsequent COVID-19 symptoms. SARS-CoV-2 enters cells by binding to the angiotensin-converting enzyme 2 (ACE2) receptor via a spike protein displayed on the viral coat surface^3,4^. The severity of the SARS-CoV-2 pandemic has initiated a major international effort to develop antibodies that bind to the SARS-CoV-2 spike protein in the ACE2 receptor binding domain (RBD). These have been derived as monoclonal antibodies (mAbs) from infected people^5–12^, by animal immunizations with SARS-CoV-2 spike glycoprotein^13–17^ or phage screening methods^18–21^. At present more than a dozen anti-SARS-CoV-2 mAbs are in clinical development^22^ of which bamlanivimab (LV-CoV555, Lilly) and a cocktail antibody containing casirivimab (REGN10933, Regeneron) and imdevimab (REGN10987, Regeneron) have received Emergency Use Approval (EUA) from the Food and Drug Administration. Etesevimab (CB6/Junshi, also known as JS-016 and LY-CoV016) is in phase I trial (NCT04441931) and is also being trialled in combination with bamlanivimab (NCT04427501). A major challenge these therapies face is the emergence of new spike protein variants with the antigenic epitopes altered to have a lower affinity to specific antibodies (variants of concern such as the so-called UK variant (B1.1.7) or South African variant (B1.351)). One of the aims of the anti-SARS-CoV-2 antibody work is the development of high affinity molecules that can bind to the RBD and prevent the virus from attaching to its cell receptor ACE2. This needs to be a rapid process as the pandemic is unabated and new variant SARS-CoV-2 viruses begin to appear^23–27^ that could potentially need a new generation of antibodies. Antibody development can be a laborious process involving affinity maturation to generate the highest affinity molecules with the most effective virus neutralization potency. Given the unprecedented manufacturing scale-up challenges that would be required to produce and rapidly treat millions of people, it is pertinent that the most effective antibodies are prioritized and developed whereby antibody dose per patient can be minimized without compromising efficacy. With this in mind, we adapted a new method^1^ for increasing valency of antibodies by fusing the human p53 tetramerization domain (TD)^28^. The multivalent antibodies created by this method are self-assembling multimeric proteins that can be engineered into various different formats^1^.This simple method can be applied to any antibody and is currently especially pertinent to anti-SARS-CoV-2 antibodies. In the case of variant SARS-CoV-2 emerging with lower affinity for a particular epitope-paratope interaction, increased valency^18^ helps to counteract this loss in affinity with an increase in avidity, potentially maintaining the neutralizing potency of the antibody. We have applied this method to six published anti-SARS-CoV-2 antibodies, two of which are currently in clinical use for COVID-19. We show that all the tetramerized antibodies have dramatically enhanced binding properties against SARS-CoV-2 spike protein. The simple multimerization strategy described in this study can be rapidly adapted to reformat other anti-SARS-CoV-2 antibodies or antibodies against any other targets. The enhanced anti-SARS-CoV-2 antibodies are now available for clinical development.

## Results

### A method for enhancement of anti-SARS-CoV-2 antibody potency by tetramerization

**Figure 1:**
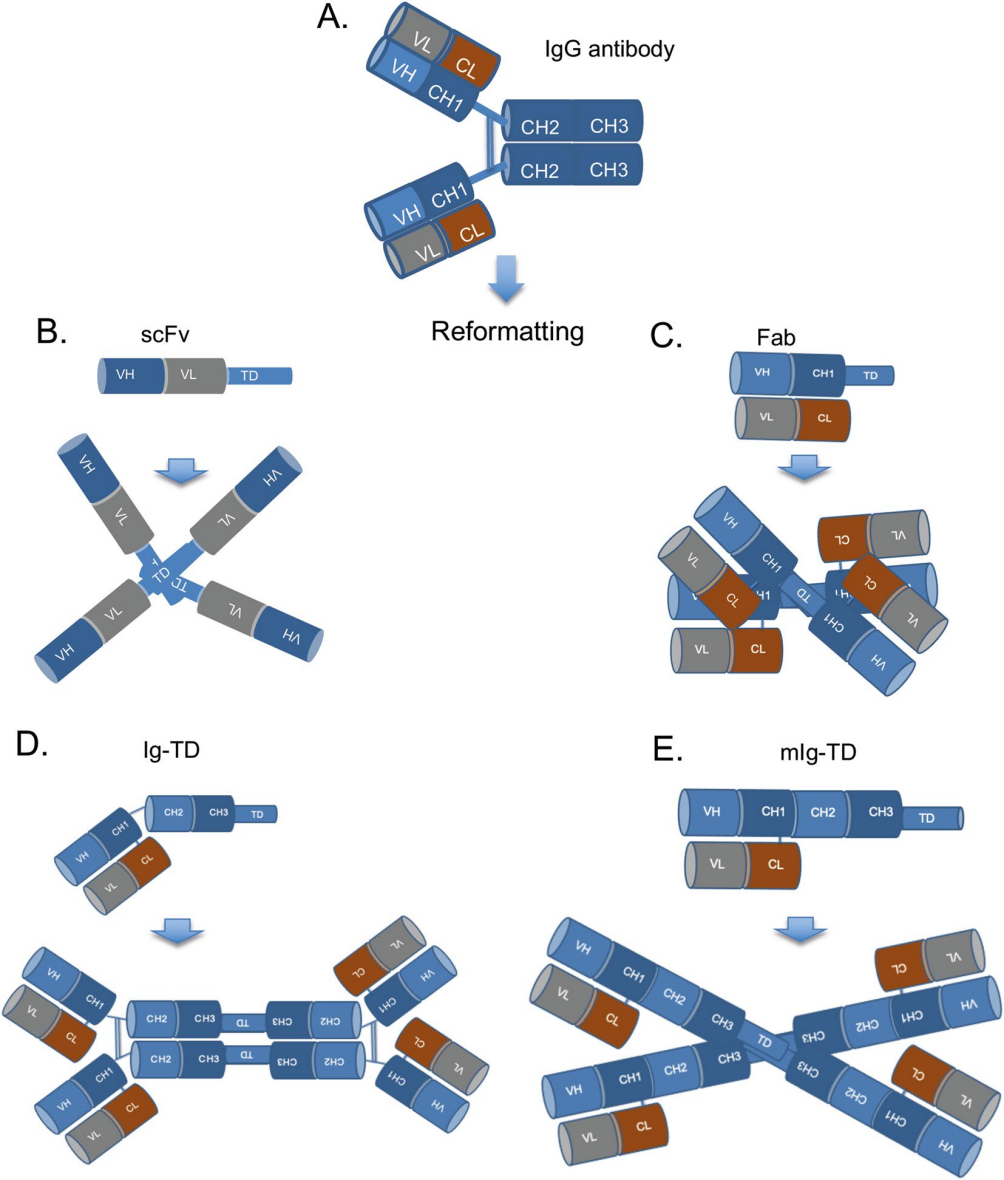
Reformatting anti-SARS-CoV-2 antibodies into tetrameric proteins. The clinical stage antibodies REGN10987, REGN10933 and CB6/Junshi are IgG1 immunoglobulins and were redesigned into various tetrameric Quad proteins. IgG1 is depicted in **(A)** showing heavy and light chain variable (V) regions, the light chain constant (C) region that is disulphide bonded to the heavy chain CH1 domain, the two heavy chains are disulphide bonded between the hinge regions. Four tetramer formats were developed. **(B)**; scFv-TD comprising VH and VL joined by a flexible linker and fused to the p53 tetramerization domain (TD). This complex is a tetravalent scFv. **(C)**: Fab-TD comprising the Fab fragment of IgG with the TD fused to the heavy chain CH1. This complex is tetravalent for the Fab. **(D)** Ig-TD comprising a heavy and a light chain with the TD fused to the heavy chain CH3. This forms a complex with the TDs and disulphide bonding between the heavy chain hinge regions. **(E)** mIg-TD is like Ig-TD but lacks the hinge region. This forms a complex with the TDs without disulphide bonding between the heavy chain hinge regions.

A method for increasing valency of antibodies^1^ was employed to reformat published anti-SARS-CoV-2 antibodies to increase the valency and augment functional affinity. The method is flexible because it simply relies on the p53 tetramerization domain to create self-multimerizing proteins. We have engineered various types of reformatted, engineered antibodies compared to IgG antibody as illustrated in Fig. 1. These include single chain variable fragment (scFv)-TD (Fig. 1B), fragment antigen-binding (Fab)-TD (Fig. 1C), whole immunoglobulin (Ig)-TD (Fig. 1D) or whole Ig but lacking the hinge regions where disulphide bonding would normally occur (monomeric, mIg)-TD (Fig. 1E).

**Figure 2:**
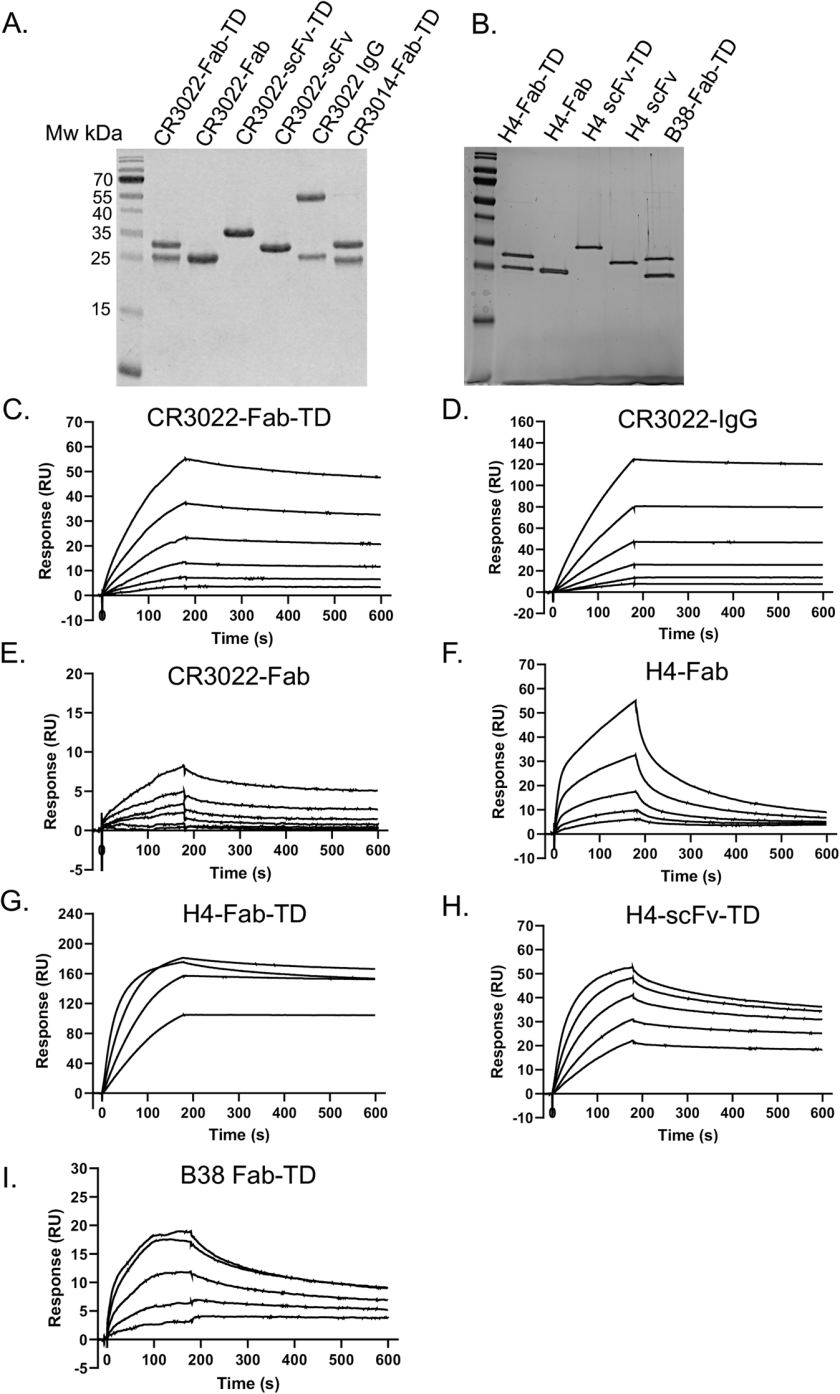
Characterization of anti-SARS-Cov-2 antibodies with different valencies by surface plasmon resonance. The binding potencies of the anti-SARS-Cov-2 tetrameric Quad proteins was determined using surface plasmon resonance. Purified proteins were captured using immobilized SARS-Cov-2 RBD, carried out with a Biacore T200 instrument. **(A)** Anti-SARS-Cov-2 antibodies were expressed by transfection and secretion from Expi293 suspension cells and characterized by SDS-PAGE following purification by Ni-affinity chromatography and gel filtration. Gels were stained with InstantBlue Ultrafast Protein Stain and Mw size markers included (left hand lanes)*.* Uncropped gels are shown in Supplementary Fig. 7. **(C–I)** Antibody binding to SARS-Cov-2 RBD was evaluated using SPR. Biotinylated SARS-CoV-2 RBD was captured on a streptavidin chip and antibodies flowed over the surface at different concentrations. CR3022 and CR3014 antibody concentrations were 10, 5, 2.5, 1.25, 0.625 and 0.312 nM, while those for H4 and B38 antibodies were 12.5, 6.25, 3.125, 1.56 and 0.78 nM (the H4-Fab-TD has four curves 6.25, 3.125, 1.56 and 0.78 nM). Sensorgrams representative of two independent experiments are shown for **(C)** CR3022-Fab-TD, **(D)** CR3022-IgG, **(E)** CR3022-Fab, **(F)** H4-Fab, **(G)** H4-Fab-TD, **(H)** H4-scFv-TD and **(I)** B38-Fab-TD. Kinetic parameters are shown in Supplementary Table S1. Tetramerization decreases dissociation rates for the multimeric species compared to the monomeric proteins. This results in increased affinity for SARS-Cov-2 RBD for CR3022-Fab-TD compared to CR3022-Fab, and for H4-Fab-TD and H4-scFv-TD compared to H4-Fab.

We initially evaluated three anti-SARS-CoV-2 antibodies, viz. CR3022 isolated from a phage library^8^ and two mAbs obtained using data from a COVID-19 convalescent patient (H4 and B38)^12^ to evaluate the effect of multimerization on binding. We used CR3014 as a negative control as it binds SARS-CoV but not SARS-CoV-2^8^. In these cases, the antibodies were prepared as tetramers with Fab (Fab-TD) or single chain variable fragment (scFv-TD) formats and the Quads purified to homogeneity by nickel chromatography The protein preparations were analyzed by size exclusion chromatography (SEC) or SDS-PAGE to confirm the component chains, lack of aggregates and mono-dispersity (Supplementary Fig. 1, Fig. 2A, B and Supplementary Fig. 2). The scFv-TD and Fab-TD mAbs quads were purified using Ni–NTA and the Fc containing Ig-TD and mIg-TD mAbs formats were purified by Protein A affinity chromatography. As the Expi293F system produces substantially clean, secreted proteins, we required limited use of SEC. The data in Supplementary Fig. 1 and Supplementary Fig. 2 show proteins respectively separated on analytical SEC or non-reducing SDS-PAGE and no evidence of aggregates was observed, In addition, in our previous work^1^, tetrameric proteins (scFv or Dabs), were compared by SEC with monomeric versions and the tetramers chromatographed as single peaks corresponding to the presence of uniform populations, without detectable aggregation.

In order to ensure maintained binding efficacy in the tetrameric forms, we evaluated the binding properties of the engineered antibodies by surface plasmon resonance (SPR) assays (Fig. 2C–I) with immobilized SARS-CoV-2 RBD protein. The k_off_ values of the bivalent CR3022 IgG format and the engineered tetravalent CR3022 Fab-TD Quad were 1.2 × 10^–4^ and 4.2 × 10^–4^ s^−1^ respectively (Supplementary Table 1A). Further, the two COVID-19 patient-derived mAbs engineered as Fab-TD tetramers (H4 and B38, Fig. 2G,I) or as H4-scFv-TD (Fig. 2F) also maintained RBD binding and displayed slow off rates. The Fab versions (both CR3022 and H4) have more rapid off rate and subsequently weaker Kd values (Fig. 2E,F). Comparing H4-Fab-TD and H4-scFv-TD with H4-Fab illustrates the improved k_off_ and subsequently K_d_ values (Fig. 2F–H, Supplementary Table 1B) that results from the increase of valency. Notably, the H4-Fab-TD out-performs the H4-scFv-TD, presumably because the tetravalent Fab has additional avidity effects on binding to the RBD on the SPR chip. The Kd of Fab version of the H4 mAb (14 nM) is poor compared to the Quad proteins, mainly due to the relatively fast k_off_ (Supplementary Table 1B). These data show that Quad proteins maintain and improve binding to the coronavirus spike protein after tetramerization. No binding to SARS-CoV-2 RBD was observed with the CR3014-Fab-TD Quad as expected^8^ (Supplementary Table 1A). Therefore, this method for tetramerization of the antibodies confers advantageous valency and avidity of binding to the SARS-CoV-2 RBD protein.

### Augmented detection of SARS-CoV-2 spike protein using reformatted tetrameric antibodies

**Figure 3:**
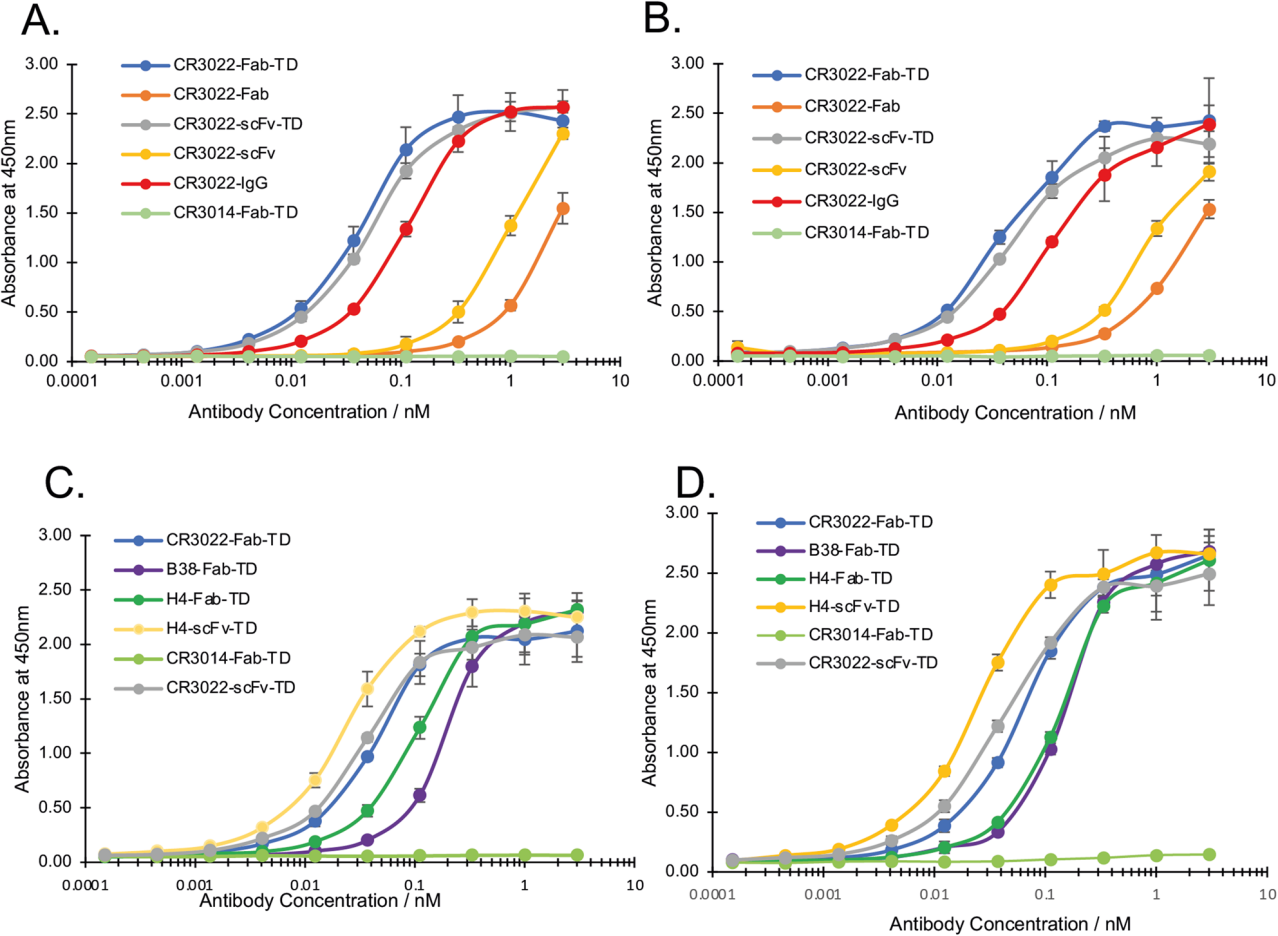
Direct ELISA immunoassays with anti-SARS-CoV-2 antibodies detecting viral spike proteins. The ability and potency of engineered anti-SARS-CoV-2 antibodies to bind to the viral spike proteins was compared using Enzyme-linked immunosorbent assays (ELISAs). SARS-CoV-2 spike protein as either S1 **(A,C)** or RBD **(B,D)** was adsorbed onto immunoassay plates at 2 µg per µL for 16 h at 4 C. Wells were thoroughly washed, blocked, and various concentrations of the indicated His-tagged antibodies were added to each well and incubated for 16 h at 4 C, followed by washing and incubation with anti-His-HRP antibody for 2 h at room temperature. Colour was developed using TMB and absorbance read at 450λ. All samples were run in triplicate.

We conducted direct enzyme-linked immunosorbent assays (ELISAs) to evaluate the effects of valency on the binding efficacy of the tetramerized Quad antibodies. We used spike protein-absorbed ELISA plates, with either SARS-CoV-2 S1 spike protein (Fig. 3A, comparing CR3022 formats and Fig. 3C comparing CR3022 with H4 and B38 Quads) or SARS-CoV-2 RBD (Fig. 3B,D with similar comparisons), and titrated antibody binding (with the His-tagged constructs detected by anti-HIS HRP antibody). CR3022-scFv or CR3022-Fab showed approximately 50% binding at about 1 nM when interacting with either S1 or RBD, whereas whole IgG is closer to 0.1 nM (Fig. 3A,B). The CR3022-Fab-TD or scFv-TD display the best functional affinity in this assay as the 50% binding of the tetramers CR3022-Fab-TD or scFv-TD was closer to 0.05 nM, due to increased valency and avidity effects of tetramerization.

The consequence of tetramerization was also tested with binding of the H4 and B38 mAbs, comparing interaction of H4-Fab-TD or H4-scFv-TD with CR3022-Fab-TD or CR3022-scFv-TD with the SARS-CoV-2 S1 spike protein (Fig. 3C) or the RBD (Fig. 3D). We observed the most potent Quad antibody was the H4-scFv-TD (50% binding about 0.02 nM) substantially better than CR3022-scFv-TD (50% binding about 0.05 nM). The H4 or B38 Fab-TD proteins did not have especially potent binding curves, displaying 50% binding at about 0.1 nM and 0.2 nM respectively.

The increase in functional affinity in the reformatted H4 and CR3022 was emphasized by comparison of their scFv-TD forms with the H4-scFv or the CR3022-scFv and of Fab-TD with CR3022-Fab (respectively Supplementary Fig. 3A,B). The Quads saturate spike protein binding at lower molarity than the scFv or Fab counterparts. The antigenic epitopes on the SARS-CoV-2 spike protein for CR3022, H4 and B38 antibodies does not overlap^12^. It was possible therefore that the enhanced mAb tetramers could give additive effects for detection of the virus protein. This was assessed in ELISA binding of mixtures of CR3022 Fab-TD, B38-Fab-TD and H4-Fab-TD to SARS-CoV-2 RBD or S1 compared to CR3022-Fab-TD alone (respectively Supplementary Fig. 3C,D). We did not observe additive effects, probably because of steric hindrance of the bulky antibody tetramers binding to the spike protein antigens in proximity.

We further evaluated the potential of employing anti-SARS-CoV-2 CR3022 mAb in viral antigen detection systems. The spike protein detection levels were determined in a sandwich ELISA using, as capture proteins, a dimeric version of the extracellular domain of SARS-CoV-2 receptor ACE2-Fc^29^ (Supplementary Fig. 3E) or a tetrameric version (ACE2-Fc-TD)^30^ (Supplementary Fig. 3F). The CR3022 Fab-TD and scFv-TD tetramers displayed the highest signal performing with higher efficacy than the CR3022 IgG1 in various situations (Supplementary Fig. 3E–H)*.* In all ELISA data, the non-binding CR3044 served as negative control, as predicted from published binding data^8^.

### Potency threshold increase in virus neutralization by tetrameric antibodies

**Figure 4:**
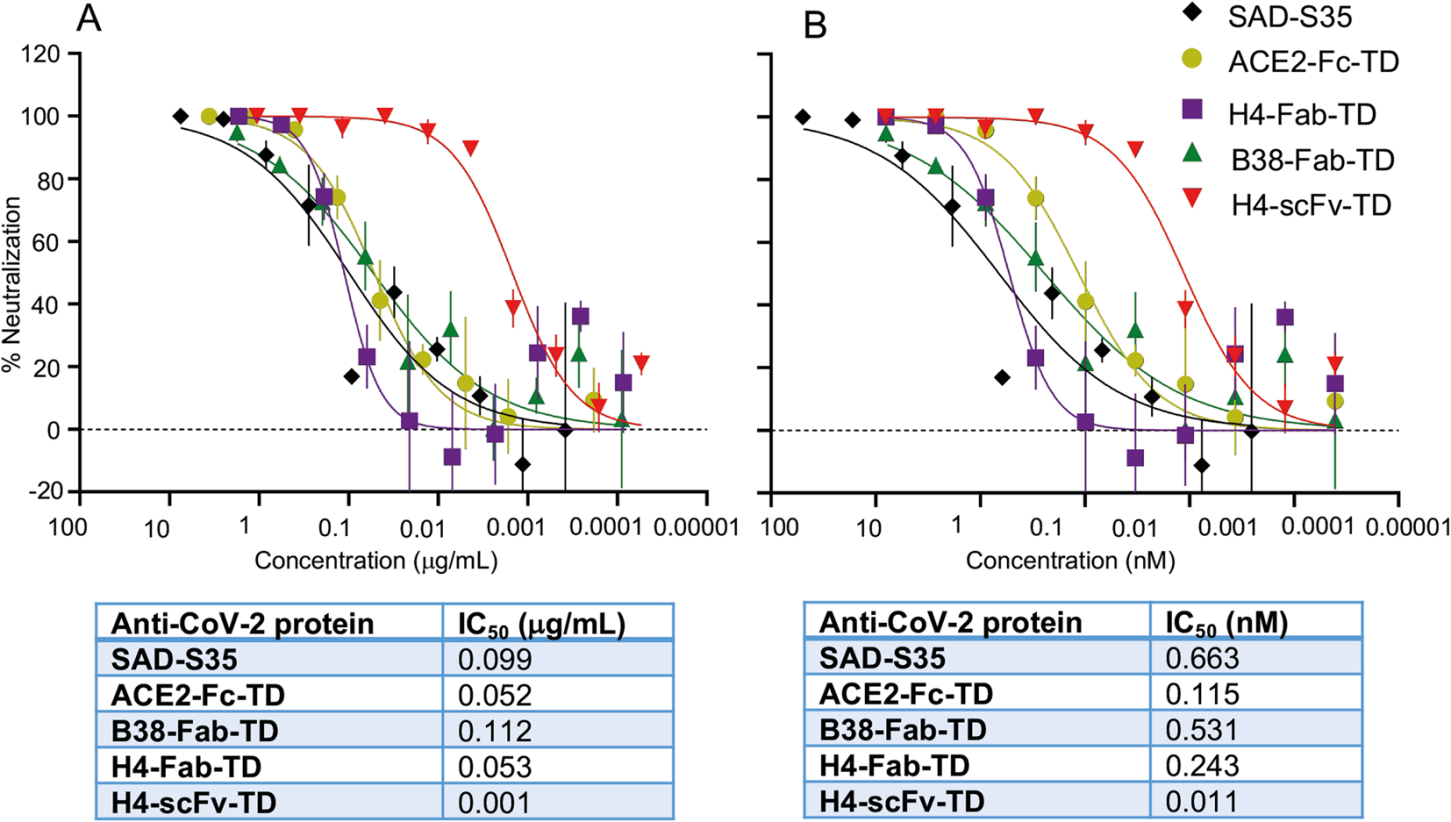
Tetramerized H4 and B38 anti-SARS-CoV-2 antibodies are potent inhibitors of virus infection. The viral infection neutralizing potency of tetrameric versions of the mAbs H4 and B38 was examined in SARS-CoV-2 pseudovirus infection assays. Increasing concentrations of H4-scFv-TD, H4-Fab-TD or B38-Fab-TD were incubated with HEK 293 T/17-A2-T2 cells after 1 h. The cells were incubated for 60 h before luciferase activity, indicative of viral entry, was determined. The efficacy was compared to the neutralization effects of a commercial anti-SARS-CoV-2 mAb SAD-S35 (AcroBiosystems) and a tetramerized ACE2-Fc-TD. Data are represented in µg/mL **(A)** and nM **(B)**. Calculated IC_50_ values are tabulated below each set of curves. The assays were performed twice, in duplicate with error bars indicating the standard error.

The H4 and B38 mAbs bind different epitopes on the spike protein and both are neutralizing^12^. The tetramer reformatted Quad versions of these were tested for their ability to interfere with coronavirus infection using a pseudotyped lentivirus-based neutralization assay. Interference with pseudotyped virus infection of HEK293T/17 cells stably expressing the ACE2 receptor and TMPRSS2 protease (HEK293T/17-A2-T2) by either H4-Fab-TD, H4-scFv-TD or B38-Fab-TD was compared to a commercially available neutralizing IgG mAb (SAD-S35) and to our tetramerized ACE2-Fc-TD protein^30^. The most effective protein in this neutralization assay was the tetrameric scFv version of H4 that interferes with a 50% inhibitory concentration (IC_50_) 0.001 µg/mL compared to 0.099 µg/mL for SAD-S35 and 0.052 µg/mL for ACE2-Fc-TD (Fig. 4A). The H4 and B38 Fab-TD antibodies have IC_50_ values of 0.053 and 0.112 µg/mL respectively (Fig. 4A). The IC_50_ of H4-scFv-TD and of ACE2-Fc-TD are closer when determined from molar amounts, being respectively 0.011 nM and 0.115 nM (Fig. 4B). These data show that the mAbs reformatted as Quad tetramers have greater potency for virus neutralization.

As a means to expand on the flexibility of the tetramer valency increase method demonstrated with Quad formats of CR3022,H4 and B38, we investigated anti-SARS-CoV-2 antibodies for which clinical trials have been recently reported. There are several such anti-SARS-CoV-2 antibodies under clinical trial investigation either as mono-therapy, for instance, LY-CoV555^7^ and CB6/Junshi, (etesevimab, also called LY-CoV016) are in phase I trials or as a cocktail REGN-COV2 which is a mixture of mAbs, REGN10987 (imdevimab) and REGN10933 (casirivimab)^31^. Accordingly, we reformatted the mAbs REGN10987, REGN10933 and CB6/Junshi with the p53 tetramerization domain into three types of Quad antibody format, namely Fab-TD, Ig-TD and mIg-TD (illustrated in Fig. 1) to compare with the IgG antibody forms that are in the clinical trials.

These Quad proteins were purified using protein A or Ni-HTA chromatography and purity and integrity assessed with SDS-PAGE in reducing and non-reducing conditions (Supplementary Fig. 2A–C). To ensure that the Quad protein formats bind to the SARS-CoV-2 RBD and to compare with the IgG forms, a plate-based surrogate neutralization assay measuring competitive binding of ACE2 with SARS-CoV-2 RBD was established (Supplementary Fig. 5). Anti-human IgG was used to capture ACE2-Fc and this was incubated with pre-mixed anti-SARS-CoV-2 competitor antibodies plus biotinylated SARS-CoV-2 RBD. Non-competed RBD that bound to ACE2-Fc was detected with HRP-conjugated streptavidin. In each case, we observed ACE2 binding neutralization was achieved at substantially lower concentrations of the tetramerized forms of mAb compared to the original IgG (Supplementary Fig. 5, panels A–C Fab-TD, panels D-F mIg-TD and panels G-I Ig-TD). The fold potency change of the multimerized mAb versions was directly compared to the IgG formats and calculated based on the IC_50_ values (Supplementary Fig. 5J,K). In this plate assay, the Fab-TD proteins were found to be consistently the most potent in neutralizing ACE2 interaction with SARS-CoV-2 RBD. The two larger molecular weight complexes of the Ig-TD and mIg-TD formats, yielded a similar fold enhancement of potency. Given all three Quad configurations are tetrameric molecules, it was interesting to observe that the size, shape and flexibility of these multimeric molecules appear to play an important role in potency modulation. Overall, the improvement trends in this plate-based analysis consistently shows that all three anti-SARS-CoV-2 Quad formats have greatly improved potency over the clinical stage mAbs.

**Figure 5:**
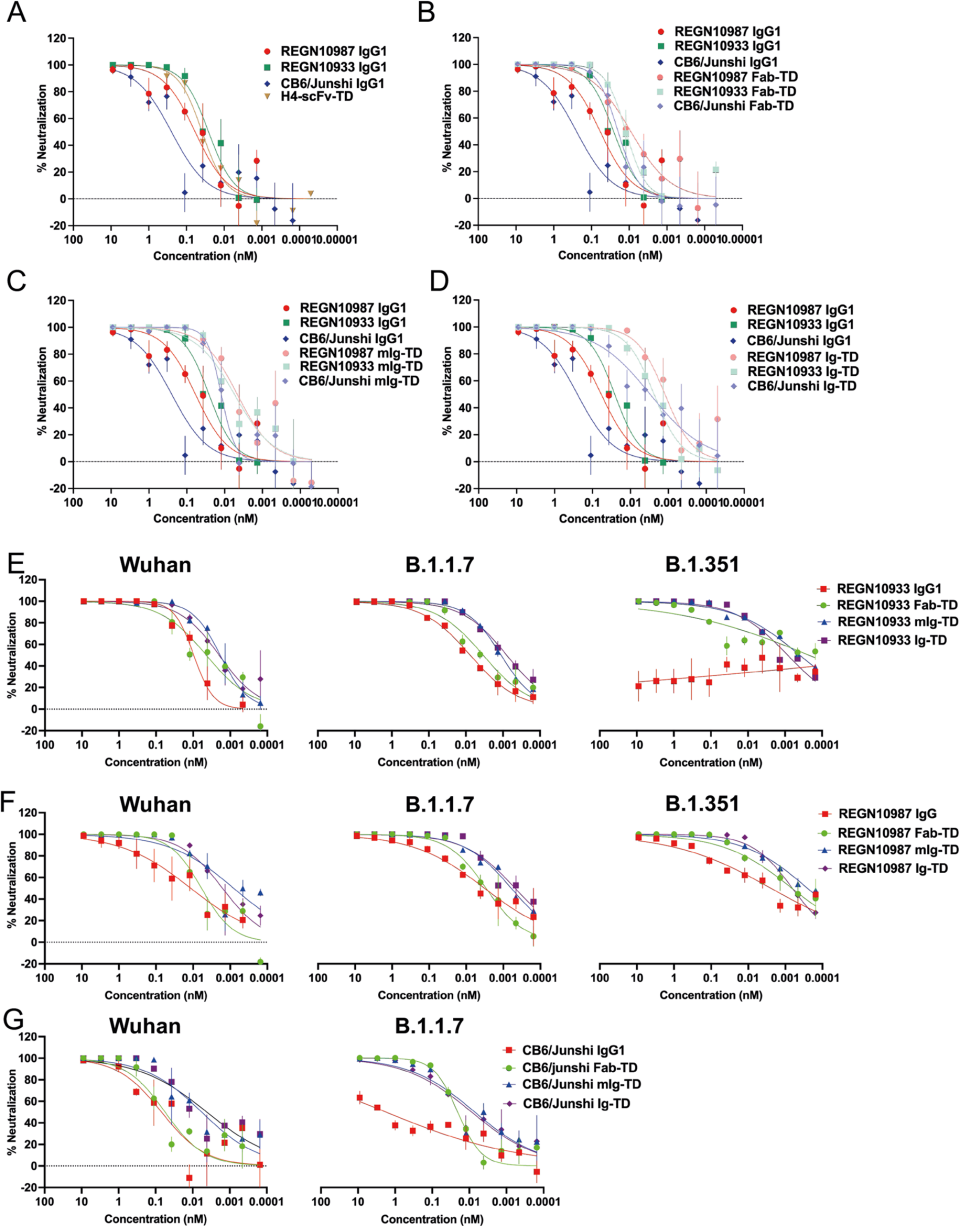
Neutralization of pseudovirus infection by tetramerized clinical stage SARS-CoV-2 antibodies. The potency of clinical stage mAbs that were engineered as tetramer formats was measured in SARS-CoV-2 pseudovirus infection assays. Increasing concentrations of antibodies were incubated with Wuhan pseudovirus for 1 h before addition to HEK 293 T/1A2-T2 cells. The cells were incubated for 60 h before luciferase activity was determined to show viral entry. The data are split to different panels for clarity. **(A)** The neutralization potency of H4-scFv-TD Quad was compared to clinical stage REGN10987, REGN10933 and CB6/Junshi IgG1. **(B)** The neutralization potency of REGN10987, REGN10933 and CB6/Junshi IgG1 was compared to REGN10987 Fab-TD, CB6/Junshi Fab-TD and REGN10933 Fab-TD. **(C)** The neutralization potency of the REGN10987, REGN10933 and CB6/Junshi IgG1 was compared to REGN10987 mIg-TD, REGN10987 mIg-TD and CB6/Junshi mIg-TD. **(D)** The neutralization potency of the REGN10987, REGN10933 and CB6/Junshi IgG1 was compared to REGN10987 Ig-TD, CB6/Junshi Ig-TD and REGN10933 Ig-TD. The assays were performed twice and each point in duplicate. The error bars indicate the standard errors. Computed IC_50_ data (nM and µg/ml) and fold change based on nM are shown in Supplementary Fig. 4. Tetramerised clinical stage mAbs potency neutralisation potency was measured against SARS-CoV-2 pseudovirus variants by increasing concentrations of antibodies incubated with Wuhan, B.1.1.7 or B.1.351 pseudoviruses. **(E)** Neutralization potency of REGN10933 IgG1, Fab-TD, mIg-TD and Ig-TD against Wuhan, B.1.1.7 and B.1.351 pseudoviruses **(F)** Neutralization potency of REGN10987 IgG1, Fab-TD, mIg-TD and Ig-TD against Wuhan, B.1.1.7 and B.1.351 pseudoviruses **(G)** The neutralization potency of CB6/Junshi IgG1, Fab-TD, mIg-TD and Ig-TD against Wuhan, B.1.1.7 and B.1.351 pseudoviruses. The assays were performed twice and each point in duplicate. The error bars indicate standard deviations.

The gold standard cell-based test for the efficacy of viral reagents is the capacity to neutralize the entry infection of viral particles into cells expressing the ACE2 receptor. We have used this approach to evaluate the H4 and B38 Quad proteins shown in Fig. 4. Accordingly, we have determined the comparative efficiency that the Quad versions of the mAbs currently being evaluated in clinical trials to neutralize pseudotyped lentiviral infection into a permissive human cell line. Aligned to the plate competition assay, we compared the REGN10987, REGN10933 and CB6/Junshi IgG with Fab-TD, mIg-TD and Ig-TD formats. In addition, we compared these with the tetravalent H4 mAb in the form of an scFv tetrameric version (H4-scFv-TD) (Fig. 5). As a bench mark, Fig. 5A displays the comparison of IgG proteins with the H4-scFv-TD showing that the two mAbs (REGN10987, REGN10933) and H4-scFv-TD have similar viral neutralization potencies. The CB6/Junshi mAb is the least potent (the calculated IC_50_ values are in Fig. 5E). The relative neutralization potencies of the mAb IgG proteins are shown compared with the engineered tetramers, respectively Fab-TD (Fig. 5B), mIg-TD (Fig. 5C) and Ig-TD (Fig. 5D) together with IC_50_ values and fold change in neutralization (Fig. 5E).

In all three tetramer formats of the mAbs, there is improved IC_50_ compared to IgG in the neutralization of the pseudoviral infection and the potency trend in this assay is the Fab-TD, mIg-TD to Ig-TD. The Ig-TDs have the best IC_50_ enhancement being 59 (REGN10987), 13 (REGN10933) and 83 (CB6/Junshi) fold change compared to IgG mAbs (Fig. 5D,E). The scFv-TD engineered version of the H4 mAb has an IC_50_ in the viral assay that approximates to those found for the Fab-TD and mIg-TD versions of the three clinical trial antibodies. Overall, we observed the greatest improvement for the CB6/Junshi tetramers and the greatest improved IC_50_ being in the CB6/Junshi Ig-TD (Fig. 5D). Interestingly, the Fab tetramers were the least potent in the viral assay (Fig. 5) compared to the plate-based competition assays (Supplementary Fig. 5).

Rapid emergence of SARS-CoV-2 variants, and especially those with spike protein mutations, is of concern to the efficacy of antibody therapeutics and to immunization. Alteration or deletion of spike epitopes can prevent neutralization by antibodies effective against variants of the original Wuhan strain. Therefore testing the neutralization potency of antibody therapeutics against variants is vitally important. To this end, we have investigated pseudoviruses bearing the mutant spikes of B.1.1.7 or B.1.351 variants of concern and tested the neutralization potency of REGN10933, REGN10987 and CB6/Junshi formats (IgG1, Fab-TD, mIg-TD and Ig-TD) against these. In general, as with the Wuhan pseudovirus neutralization, Quad formats of REGN10933, REGN10987 and CB6/Junshi (Fig. 5E–G respectively) showed more potent neutralization than the bivalent IgG formats. While REGN10933 IgG1 lost all neutralizing activity against B.1.351, the TD formats (and particularly the mIg-TD and Ig-TD formats) retained neutralizing activity (Fig. 5E). The quad formats of REGN10987 were also observed to be more potent than the IgG1 format against the B.1.1.7 and B.1.351 variants (Fig. 5F). The CB6/Junshi IgG1 showed weak potency against the B.1.1.7 variant, but again the quad formats retained potency. Notably the mutations present in the B.1.351 spike render CB6/Junshi non-neutralizing across all formats (Fig. 5G). Fold improvements in IC_50_ for the Ig-TD vs IgG1 against the B.1.1.7 variant ranged from 9 to more than 200-fold (Supplementary Fig. 5). Interestingly the mIg-TD format of CB6/Junshi was more slightly more potent than the Ig-TD format against the B.1.1.7 variant (Fig. 5G). The same feature was observed more strongly for the REGN10933 and REGN10987 mIg-TD and Ig-TD formats against the B.1.351 variant (Fig. 5F).

## Discussion

### A simple protein engineering method to increase valency and avidity of anti-SARS-CoV-2 antibodies

We show that implementing the tetramerization method generates antibodies with dramatically enhanced binding and importantly significantly enhanced neutralization properties against SARS-CoV-2 spike protein. These super-charged multivalent anti-SARS-CoV-2 mAbs are now available for further characterization and clinical investigation as the next-generation of anti-SARS-CoV-2 antibodies with superior binding and neutralization potency. This implies that lower dosage than currently used could be employed for therapeutic responses, which would be valuable for each treatment bolus but also for mass production as necessary amounts would be substantially lowered. Therefore, they have the potential to be developed as more effective treatment based on milligram scale doses as opposed to the gram-scale doses seen in the current EUA monoclonal antibody treatments^7,31^.

Our findings are supporting evidence that increased antibody valency is a potent weapon against SARS CoV-2, and particularly in allowing antibody therapies to circumvent spike mutations which decrease the affinity of a particular paratope-epitope interaction. Our multivalent antibody method is a rapid technology that can boost antibody binding akin to affinity maturation. The method could easily be applied to other published anti-SARS-CoV-2 antibodies because it only needs inclusion of the p53 tetramerization domain. Further, the predicted structure of the Ig-TD, as we also noted for our tetrameric ACE2 protein^30^, is analogous to the structure of IgA antibodies which are tetrameric due to two IgA monomers being linked by the J chain. This IgA analogy may explain the potency enhancement in viral neutralization because potentially the Ig-TD can engage more than one viral particle, while the mIg-TD may be structurally constrained to function in the same way. Addition of the p53 tetramerization domain is expected to be non-immunogenic as it is fully human and buried in a central core surrounded by antibody domains derived from human antibodies.

### Super-charged antibodies with enhanced neutralization potency through multimerization

There is a remarkable increase in neutralization potency of the tetramerized formats with the Ig-TD versions having up to 80-fold change in virus neutralization compared to the IgG1 mAbs. Generating high potency antibodies can be slow for a number of technical reasons and the requirement for rapid and flexible methods for enhancement is required. Several anti-SARS-CoV-2 antibodies have been developed that show extremely promising efficacy as COVID-19 therapeutic reagents^11,31^. Three of these have been subject to the tetramerization method within this study and in all three, substantial increased potency has been achieved. By demonstrating such a benefit for these clinical stage reagents, it is hoped to speed the application of this technology to current and future pandemics. As tetramerization yielded increased potency for B38 and H4, a wider variety of tetrameric formats was explored for the clinical trial antibodies to find those Quad formats with the greatest increase in potency. Thus, using this simple tetramerization method enhanced viral neutralization can be achieved due to increased valency and avidity.

A difference in the relative affinities of the H4-scFv-TD and H4-Fab-TD constructs is observed between SPR (Fig. 2G and H, H4-Fab-TD having higher affinity) and direct ELISAs (Fig. 3C,D, H4-scFv-TD having higher affinity). This could be due to factors concerning the viral spike or SARS-CoV-2 RBD coated surfaces such as surface roughness, spike/RBD density and chemical nature of spike/RBD association (e.g. specific streptavidin/biotin interaction for SPR versus stochastic adsorption for the ELISA plate surface). It is remarkable that the Ig-TD format displays the greatest effective potency on the viral interference assays (Fig. 5) while the Fab-TD formats perform better in the in vitro competition assays (Supplementary Fig. 5). This difference may be influenced by size and flexibility of the Quad proteins but also due to the antigen availability in the two assays. In the plate assay, the ACE2 protein interactions with the SARS-CoV-2 spike RBD in a two-dimensional system and the tetramer antibodies bind the SARS-CoV-2 RBD in solution. In the virus neutralization assays, the extended putative structure of the Ig-TD (like IgA) could reach between virus particles extending the potency. It is also likely that steric hindrance effects may play distinct roles in the plate assay compared to the virus-based assay. Notably, the mIg-TD format shows the highest potency against the B.1.351 pseudovirus variant (Fig. 5E–G). Perhaps some difference in the structural properties of this format better enable its interaction with the B.1.351 spike. Nonetheless, the choice of Quad format that appears to be more suitable for therapeutic development is the Ig-TD (or mIg-TD).

For potential clinical implementation, the Ig-TD (or mIg-TD) that includes intact Fc regions, would have additional advantages, as it would be able to interact with Fc-receptors and invoke Fc-mediated effector function thus providing additional functionality. Further, the Fc would also be able to engage with neonatal Fc-receptor and could promote antibody recycling through rescue from normal lysosomal degradation and thus prolonged circulation half-life.

Finally, the standard human IgG form of antibodies has a bivalent interaction with antigens. Increasing the valency of antibodies by tetramerization^1^ ,or by other multivalency methods^18,32–34^, leads to higher functional affinity through avidity effects^1,35^. The implementation of our protein engineering method using the human p53 protein tetramerization domain is a simple and flexible method to increase valency^1,30^. Since community transmission is a major problem with SARS-CoV-2 and the appearance of new variants (such as B.1.1.7 and B.1.351) that appear to have increased transmissibility^23,24,26,27^ there is a need for effective treatments alongside rapid and sensitive testing. As well as increasing potency against current variants of concern, Quad formats of existing antibodies could extend the range of rapidly emerging future variants to which they are effective. Recent successes with prophylactic therapy (NCT04452318), indicate a setting in which widespread anti-SARS-CoV-2 antibody administration could be beneficial. The increased potency and variant resistance of Quad formatted antibodies would be of great help in this setting. Employing the enhanced affinity tetrameric antibodies could be a valuable asset for lateral flow device development with reformatted antibodies that could produce enhanced signal and providing more sensitive reagents to be employed in lateral flow tests for virus.

In conclusion, our results add to the mounting evidence for increasing antibody valency for more effective therapies against SARS-CoV-2^18,35^, both in its current and future forms. We show that our method of antibody tetramerization permits reformatting of existing anti-SARS-CoV-2 antibodies for improved therapeutic and viral detection applications. The data further generalize the concept that mAbs reformatted as tetramers, using our flexible technology, will have greater potency. The technology can be applied to any antibody but also to all new forms of proteins, as we have shown for tetramerized ACE2 receptor for SARS-CoV-2^30^. It has recently been found that mRNA vaccine-induced antibodies made into monoclonal antibodies have reduced (or have lost) activity to variant SARS-CoV-2^36^. The use of tetramerization of monoclonal antibodies is one method that could be invoked to avoid such lowering or loss of efficacy as new variants arise. This will confer distinct advantages by invoking increased avidity for currently known viral targets, new viruses that may emerge in the future but also generally to any antigenic target.

## Materials and methods

### Production and characterization of recombinant proteins

Anti-SARS-CoV-2 antibodies, antibody fragments and ACE2 proteins were based on published data for these molecules^30^. Gene synthesis was used to generate clones for secretion and the human p53 tetramerization domain where appropriate for production of tetrameric Quads. All the sequences were cloned in pTT5^37^ and expressed by secretion from Expi293F (Thermofisher) according to the manufacturer’s recommendations. and the proteins were purified by nickel affinity chromatography and size exclusion as described^30^. SARS-CoV-2 viral spike protein and receptor binding protein was made as described elsewhere^30^ as Avi-tagged proteins and biotinylated using BirA enzyme^38^. Antibody proteins were characterized by SDS-PAGE and/or by size exclusion chromatography. Constructs made here produced a range of antibody protein from about 10–50 mg/L of culture.

### Cloning, protein expression and purification of multivalent formats of anti-SARS-CoV-2 clinical trial mAbs

Sequences of the Fab portion of the clinical stage mAbs REGN10987, REGN10933^31^. and CB6/Junshi^11^. were reformatted into three different tetravalent types (sequences in Supplementary Fig. 6). Construction of the Fab-TD formats involved sub-cloning the p53 tetramerization domain (TD) linked onto the C-terminus of the immunoglobulin heavy chain CH1 domain devoid of the CH2-CH3 domains. For the Ig-TD format, the TD domain was linked directly to the C-terminus of the CH3 domain. The design of the monomeric Ig-TD (mIg-TD) format was identical to the Ig-TD format except the heavy chain core hinge region was removed. The corresponding light chains (LC) of the parental mAbs were kept intact. Expression constructs containing the modified heavy chains (HC) together with the corresponding LC constructs were expressed in Expi293F cells using Expifectamine293 Reagent according to the manufacturer’s recommendations (Thermo Fisher Scientific). Multimerized mAbs were purified directly from the culture supernatant by affinity purification. Fab-TD mAbs formats were purified using Ni–NTA and the Fc containing Ig-TD and mIg-TD mAbs formats were purified by Protein A affinity chromatography. All proteins were buffer exchanged and concentrated into PBS buffer using Amicon columns (Millipore) and aliquots were stored at 4 °C or at -80 °C for long-term storage.

### Surface plasmon resonance of anti-SARS-Cov-2 antibodies with SARS-Cov-2 RBD

SPR experiments were carried out using Biacore T100 (Cytiva, formerly GE Healthcare) in PBS buffer, pH 7.4 containing 0.05% Tween-20. A streptavidin-coated SA chip (Cytiva) was washed with 3 × 30 s injections of 1 M NaCl/50 mM NaOH at 10µL/min before biotinylated SARS-Cov-2 RBD (0.2 µg/mL) was captured to target immobilisation level 500RU. Antibody binding to SARS-Cov-2 RBD was analysed at 25ºC using multi-cycle injections of antibody at six concentrations at 30µL/min for 180 s, followed by 420 s dissociation time. Concentrations between 0.312 – 10 nM were used for CR3022 and CR3014 antibodies, while concentrations between 0.78 – 12.5 nM were used for analysis of H4 and B38 antibodies. The surface was regenerated after each antibody injection using 1 M NaCl/50 mM NaOH at 30µL/min for 20 s contact time followed by 120 s stabilisation time. Flow cell 1 was used as a reference and so did not contain any captured SARS-Cov-2 RBD and was subtracted from all sensograms before analysis. Data were fitted to a 1:1 kinetics model using Biacore T200 Evaluation Software version 2.0.

### Direct ELISA methods

ELISAs were carried out in clear, flat-bottomed 96-well MaxiSorp plates obtained from Thermo-Fisher Scientific,UK. SARS-CoV-2 protein (either spike or RBD) was prepared at 2 µg per mL in PBS and 100 µL per well added to an ELISA plate alongside negative control wells with 100 µL PBS alone. Plates were then incubated for 16 h at 4 C and washed three times with 0.05% v/v Tween-20 in PBS (PBS-T) prior to addition of 150 µL of 5% w/v BSA dissolved in PBS and room temperature (RT) incubation for 4 h. Blocked wells were then washed 2 times with PBS-T, followed by the addition of 100 µL of His-tagged antibody diluted in PBS-T and the plate incubated for 16 h at 4 C. Wells were then washed 4 times with PBS-T and incubated with 100 µL of anti-His-HRP (mouse monoclonal anti-polyhistidine antibody (clone HIS-1) conjugated to peroxidase (anti-His-HRP) was obtained from Sigma-Aldrich) diluted 1:4000 with 1% w/v BSA in PBS for 2 h at RT, followed by washing 4 times with PBS-T. 25 µL of TMB was added to each well, incubated for 20 min at RT, then 25 µL 3 M HCl added and absorbance read at 450λ.

### Sandwich ELISA methods

ACE2-Fc or ACE2-Fc-TD was prepared at 3 µg per mL in PBS and 100 µL per well added to an ELISA plate alongside negative control wells with 100 µL PBS alone. Plates were then incubated for 16 h at 4 C and washed three times with 0.05% v/v Tween-20 in PBS (PBS-T) prior to addition of 150 µL of 5% w/v BSA dissolved in PBS and room temperature (RT) incubation for 4 h. Blocked wells were then washed 2 times with PBS-T, 100 µL of 0–300 nM RBD diluted in PBS-T added and the plate incubated for 16 h at 4 C, before wells were again washed 4 times with PBS-T. His-tagged antibodies diluted to 1 nM in PBS-T were then added and incubated for 3 h at RT. Wells were then washed 4 times with PBS-T and incubated with 100 µL of anti-His-HRP diluted 1:4000 with 1% w/v BSA in PBS for 2 h at RT, followed by washing 4 times with PBS-T. 25 µL of TMB was added to each well, incubated for 20 min at RT, then 25 µL 3 M HCl added and absorbance read at 450λ.

### Time course ELISA methods

ACE2-Fc-TD was prepared at 3 µg per mL in PBS and 100 µL per well added to an ELISA plate alongside negative control wells with 100 µL PBS alone. Plates were incubated for 16 h at 4 C and washed three times with 0.05% v/v Tween-20 in PBS (PBS-T) prior to addition of 150 µL of 5% w/v BSA dissolved in PBS and room temperature (RT) incubation for 4 h. Blocked wells were then washed 2 times with PBS-T, 100 µL of 0–300 nM RBD diluted in PBS-T added and the plate incubated for 10 min or 1 h at RT or 37 °C, before wells were washed 4 times with PBS-T. His-tagged CR3022-Fab-TD diluted to 1 nM in PBS-T was added and the plate incubated for 10 min or 1 h at RT or 37 °C, before wells were washed 4 times with PBS-T. 100 µL of anti-His-HRP diluted 1:4000 with 1% w/v BSA in PBS was added and the plate incubated for 10 min or 1 h at RT or 37 °C, before wells were washed 4 times with PBS-T. 25 µL of TMB was added to each well, incubated for 15 min at RT, then 25 µL 3 M HCl added and absorbance read at 450λ. For simultaneous addition experiments, the SARS-CoV-2 RBD, CR3022-Fab-TD and anti-His-HRP antibody were added together.

### Competitive ELISA with tetravalent mAb formats.

As a surrogate neutralization assay, inhibition of ACE2 interaction with SARS-CoV-2 RBD was measured using a competitive ELISA protocol. High binding 96-well plates (Corning) were coated overnight at 4 °C with 100 ng of anti-human IgG (Binding Site). Three washes were used between each subsequent step using PBS containing 0.1% Tween 20 and incubation at room temperature for 1 h. Coated ELISA plates were blocked with 1% BSA followed by the addition of 100 ng of human ACE2-Fc. A mixture containing serially diluted anti-SARS-CoV-2 multivalent mAbs starting at 30 nM and a fixed amount of biotinylated SARS-CoV-2 RBD (75 pM) was pre-incubated at room temperature for 30 min prior to adding the mixture to the coated plate. Wells containing only the biotinylated RBD with no anti-SARS-CoV-2 antibody or wells containing ELISA assay buffer only (0.1% BSA) were used as control for calculating percentage neutralization. In the next step, HRP conjugated streptavidin (Abcam) diluted at 1 in 15,000 fold was added followed by 100 µl of TMB to generate the signal. The reaction was stopped using 50 ul of 1 M sulphuric acid and the absorbance was measured at 450 nm using a CLARIOstar micro-plate reader (BMG Labtech).

### SARS-CoV-2 lentiviral pseudotyped virus neutralization assay

SARS-CoV-2 pseudotyped lentivirus was produced inHEK293T/17 cells following a similar approach as described^39^ https://doi.org/10.6084/m9.figshare.13502580^40^, a firefly luciferase reporter gene (pCSFLW) and the SARS-CoV-2 Spike gene (pCAGGS SARS-2-Spike) to produce virus containing supernatants. Neutralizing activity of antibodies was determined with 200 TCID50 of SARS-CoV-2 lentiviral pseudotyped virus using HEK293T/17 cells stably expressing ACE-2 and TMPRSS2 (HEK293T/17-A2-T2). These were plated at 20,000 cells/well in a 96-well plate for at least 2 h at 37 C, 5% CO_2_ before use. Mixtures of antibody and pseudotyped virus were incubated with target cells for 60 h at 37 C, 5% CO_2_ and infection determined by luciferase expression using the Promega Bright-Glo assay system and GloMax Navigator plate reader, following manufacturer’s instructions. Data are normalized to pseudotyped virus and cell only controls and non-linear regression analysis performed within GraphPad Prism, for IC_50_ values.

## Supplementary Information

Supplementary Information.

## References

[1] Miller A, Carr S, Rabbitts T, Ali H (2020). Multimeric antibodies with increased valency surpassing functional affinity and potency thresholds using novel formats. MAbs.

[2] Dhama, K., Patel, S.K., Sharun, K., Pathak, M., Tiwari, R., Yatoo, M.I., Malik, Y.S., Sah, R., Rabaan, A.A., Panwar, P.K., Singh, K.P., Michalak, I., Chaicumpa, W., Martinez-Pulgarin, D.F., Bonilla-Aldana, D.K. & Rodriguez-Morales, A.J. SARS-CoV-2 jumping the species barrier: Zoonotic lessons from SARS, MERS and recent advances to combat this pandemic virus*.**Travel Med. Infect. Dis.* **37**, 101830 (2020). 10.1016/j.tmaid.2020.101830 PMC7396141 32755673

[3] Kuba K (2005). A crucial role of angiotensin converting enzyme 2 (ACE2) in SARS coronavirus-induced lung injury. Nat. Med..

[4] Hoffmann, M., Kleine-Weber, H., Schroeder, S., Kruger, N., Herrler, T., Erichsen, S., Schiergens, T.S., Herrler, G., Wu, N.H., Nitsche, A., Muller, M.A., Drosten, C., & Pohlmann, S. SARS-CoV-2 cell entry depends on ACE2 and TMPRSS2 and is blocked by a clinically proven protease inhibitor*.**Cell* **181**, 271–280 e8 (2020). 10.1016/j.cell.2020.02.052 PMC7102627 32142651

[5] Brouwer PJM (2020). Potent neutralizing antibodies from COVID-19 patients define multiple targets of vulnerability. Science.

[6] Cao, Y., *et al*. Potent neutralizing antibodies against SARS-CoV-2 identified by high-throughput single-cell sequencing of convalescent patients' B cells*.**Cell* **182**, 73–84 e16 (2020). 10.1016/j.cell.2020.05.025 PMC7231725 32425270

[7] Chen, P. *et al*. SARS-CoV-2 neutralizing antibody LY-CoV555 in outpatients with Covid-19*.**N. Engl. J. Med.* (2020). 10.1056/NEJMoa2029849 PMC7646625 33113295

[8] ter Meulen, J., van den Brink, E.N., Poon, L.L., Marissen, W.E., Leung, C.S., Cox, F., Cheung, C.Y., Bakker, A.Q., Bogaards, J.A., van Deventer, E., Preiser, W., Doerr, H.W., Chow, V.T., de Kruif, J., Peiris, J.S. & Goudsmit, J. Human monoclonal antibody combination against SARS coronavirus: Synergy and coverage of escape mutants*.**PLoS Med.* **3**, e237 (2006). 10.1371/journal.pmed.0030237 PMC1483912 16796401

[9] Rogers TF (2020). Isolation of potent SARS-CoV-2 neutralizing antibodies and protection from disease in a small animal model. Science.

[10] Seydoux, E. *et al*. Analysis of a SARS-CoV-2-infected individual reveals development of potent neutralizing antibodies with limited somatic mutation*.**Immunity* **53**, 98–105 e5 (2020). 10.1016/j.immuni.2020.06.001 PMC7276322 32561270

[11] Shi R (2020). A human neutralizing antibody targets the receptor-binding site of SARS-CoV-2. Nature.

[12] Wu Y (2020). A noncompeting pair of human neutralizing antibodies block COVID-19 virus binding to its receptor ACE2. Science.

[13] Esparza TJ, Martin NP, Anderson GP, Goldman ER, Brody DL (2020). High affinity nanobodies block SARS-CoV-2 spike receptor binding domain interaction with human angiotensin converting enzyme. Sci. Rep..

[14] Hansen J (2020). Studies in humanized mice and convalescent humans yield a SARS-CoV-2 antibody cocktail. Science.

[15] Huo J (2020). Neutralizing nanobodies bind SARS-CoV-2 spike RBD and block interaction with ACE2. Nat. Struct. Mol. Biol..

[16] Wrapp, D., De Vlieger, D., Corbett, K.S., Torres, G.M., Wang, N., Van Breedam, W., Roose, K., van Schie, L., V.-C.C.-R. Team, Hoffmann, M., Pohlmann, S., Graham, B.S., Callewaert, N., Schepens, B., Saelens, X. & McLellan, J.S. Structural basis for potent neutralization of betacoronaviruses by single-domain camelid antibodies*.**Cell* **181**, 1004–1015 e15 (2020). 10.1016/j.cell.2020.04.031 PMC7199733 32375025

[17] Xiang Y, Nambulli S, Xiao Z, Liu H, Sang Z, Duprex WP, Schneidman-Duhovny D, Zhang C, Shi Y (2020). Versatile and multivalent nanobodies efficiently neutralize SARS-CoV-2. Science.

[18] Bracken, C.J., Lim, S.A., Solomon, P., Rettko, N.J., Nguyen, D.P., Zha, B.S., Schaefer, K., Byrnes, J.R., Zhou, J., Lui, I., Liu, J., Pance, K., Q.S.B. Consortium, Zhou, X.X., Leung, K.K. & Wells, J.A. Bi-paratopic and multivalent VH domains block ACE2 binding and neutralize SARS-CoV-2*.**Nat. Chem. Biol.* **17**, 113–121 (2021). 10.1038/s41589-020-00679-1 PMC8356808 33082574

[19] Custodio TF, Das H, Sheward DJ, Hanke L, Pazicky S, Pieprzyk J, Sorgenfrei M, Schroer MA, Gruzinov AY, Jeffries CM, Graewert MA, Svergun DI, Dobrev N, Remans K, Seeger MA, McInerney GM, Murrell B, Hallberg BM, Low C (2020). Selection, biophysical and structural analysis of synthetic nanobodies that effectively neutralize SARS-CoV-2. Nat. Commun..

[20] Li, W., *et al*. High potency of a bivalent human VH domain in SARS-CoV-2 animal models*.**Cell* **183**, 429–441 e16 (2020). 10.1016/j.cell.2020.09.007 PMC7473018 32941803

[21] Schoof M (2020). An ultrapotent synthetic nanobody neutralizes SARS-CoV-2 by stabilizing inactive Spike. Science.

[22] DeFrancesco L (2020). COVID-19 antibodies on trial. Nat. Biotechnol..

[23] Greaney, A.J., *et al*. Complete mapping of mutations to the SARS-CoV-2 spike receptor-binding domain that escape antibody recognition*.**bioRxiv* (2020). 10.1016/j.chom.2020.11.007 PMC7676316 33259788

[24] Hodcroft, E.B., Zuber, M., Nadeau, S., Crawford, K.H.D., Bloom, J.D., Veesler, D., Vaughan, T.G., Comas, I., Candelas, F.G., Stadler, T. & Neher, R.A. Emergence and spread of a SARS-CoV-2 variant through Europe in the summer of 2020*.**medRxiv* (2020).

[25] Kemp, S.A. *et al*. Neutralising antibodies in spike mediated SARS-CoV-2 adaptation*.**medRxiv* (2020).

[26] Korber, B. *et al*. Tracking changes in SARS-CoV-2 spike: Evidence that D614G increases infectivity of the COVID-19 virus*.**Cell* **182**, 812–827 e19 (2020). 10.1016/j.cell.2020.06.043 PMC7332439 32697968

[27] Popa, A. *et al.* Genomic epidemiology of superspreading events in Austria reveals mutational dynamics and transmission properties of SARS-CoV-2*.**Sci. Transl. Med.* **12** (2020). 10.1126/scitranslmed.abe2555 PMC7857414 33229462

[28] Melero R, Rajagopalan S, Lazaro M, Joerger AC, Brandt T, Veprintsev DB, Lasso G, Gil D, Scheres SH, Carazo JM, Fersht AR, Valle M (2011). Electron microscopy studies on the quaternary structure of p53 reveal different binding modes for p53 tetramers in complex with DNA. Proc. Natl. Acad. Sci. U S A.

[29] Lei C, Qian K, Li T, Zhang S, Fu W, Ding M, Hu S (2020). Neutralization of SARS-CoV-2 spike pseudotyped virus by recombinant ACE2-Ig. Nat. Commun..

[30] Miller, A., Leach, A., Thomas, J., McAndrew, C., Bentley, E., Mattiuzzo, G., John, L., Mirazimi, A., Harris, G., Gamage, N., Carr, S., Ali, H., van Montfort, R.L. & Rabbitts, T.H. A super-potent tetramerized ACE2 protein displays enhanced neutralization of SARS-CoV-2 virus infection. (2021) **(submitted for publication)**. 10.1038/s41598-021-89957-z PMC8134500 34012108

[31] Weinreich, D.M. *et al*. REGN-COV2, a neutralizing antibody cocktail, in outpatients with Covid-19*.**N. Engl. J. Med.* (2020). 10.1056/NEJMoa2035002 PMC7781102 33332778

[32] Fan CY, Huang CC, Chiu WC, Lai CC, Liou GG, Li HC, Chou MY (2008). Production of multivalent protein binders using a self-trimerizing collagen-like peptide scaffold. FASEB J..

[33] Liu M, Wang X, Yin C, Zhang Z, Lin Q, Zhen Y, Huang H (2007). Targeting TNF-alpha with a tetravalent mini-antibody TNF-TeAb. Biochem. J..

[34] Zhu X, Wang L, Liu R, Flutter B, Li S, Ding J, Tao H, Liu C, Sun M, Gao B (2010). COMBODY: One-domain antibody multimer with improved avidity. Immunol. Cell Biol..

[35] Liu, H., Wu, N.C., Yuan, M., Bangaru, S., Torres, J.L., Caniels, T.G., van Schooten, J., Zhu, X., Lee, C.D., Brouwer, P.J.M., van Gils, M.J., Sanders, R.W., Ward, A.B. & Wilson, I.A. Cross-neutralization of a SARS-CoV-2 antibody to a functionally conserved site is mediated by avidity*.**Immunity* **53**, 1272–1280 e5 (2020). 10.1016/j.immuni.2020.10.023 PMC7687367 33242394

[36] Wang, Z. *et al*. mRNA vaccine-elicited antibodies to SARS-CoV-2 and circulating variants*.**Nature* (2021). 10.1038/s41586-021-03324-6 PMC8503938 33567448

[37] Durocher Y, Perret S, Kamen A (2002). High-level and high-throughput recombinant protein production by transient transfection of suspension-growing human 293-EBNA1 cells. Nucleic Acids Res..

[38] Fairhead M, Howarth M (2015). Site-specific biotinylation of purified proteins using BirA. Methods Mol. Biol..

[39] Carnell, G., Grehan, K., Ferrera, F., Molesti, E. & Temperton, N. An optimized method for the production using PEI, tritration and neutralization of SARS-CoV spike luciferase pseudotypes*.**Bio-protocol* **7**, e2514 (2017). 10.21769/BioProtoc.2514 PMC8413606 34541175

[40] Zufferey R, Nagy D, Mandel RJ, Naldini L, Trono D (1997). Multiply attenuated lentiviral vector achieves efficient gene delivery in vivo. Nat. Biotechnol..

