# Supplementary material for: Implementing a method for engineering multivalency to substantially enhance binding of clinical trial anti-SARS-CoV-2 antibodies to wildtype spike and variants of concern proteins
Source: Sci Rep. 2021 May 18;11:10475. doi: 10.1038/s41598-021-89887-w (PMC8131632; doi:10.1038/s41598-021-89887-w)
Supplement: Supplementary file 1 — Supplementary Information. [file 41598_2021_89887_MOESM1_ESM.pdf]

Implementing a method for engineering multivalent antibodies to substantially enhance functional affinity of clinical trial anti-SARS-CoV-2 antibodies

Adam Leach, Ami Miller, Emma Bentley, Giada Mattiuzzo, Jemima Thomas, Craig McAndrew, Rob Van Montfort, Terence Rabbitts

Supplementary Table 1.

A	K_d (nM)	k_{on} ($M^{-1}s^{-1}$)	k_{off} (s^{-1})
**CR3022-Fab-TD**	0.314 ±0.023	$1.34 \times 10^6 \pm 0.54 \times 10^6$	0.00042 ±0.00014
**CR3022IgG**	0.076 ±0.014	$1.69 \times 10^6 \pm 1.35 \times 10^6$	0.00012 ±0.00008
**CR3022-Fab**	2.93 ±0.95	$0.64 \times 10^6 \pm 0.50 \times 10^6$	0.00162 ±0.00087
**CR3014-Fab-TD**	No binding observed	No binding observed	No binding observed

B	K_d (nM)	k_{on} ($M^{-1}s^{-1}$)	k_{off} (s^{-1})
**H4-Fab-TD**	0.039 ±0.035	$12.1 \times 10^6 \pm 3.42 \times 10^6$	0.00042 ±0.00029
**H4-scFv-TD**	0.135 ±0.033	$2.93 \times 10^6 \pm 1.70 \times 10^6$	0.00042 ±0.00033
**H4-Fab**	14 ±11	$1.95 \times 10^6 \pm 1.87 \times 10^6$	0.01537 ±0.00542
**B38-Fab-TD**	0.327 ±0.208	$3.96 \times 10^6 \pm 1.28 \times 10^6$	0.00115 ±0.00036

Supplementary Table 1. Binding parameters for anti-SARS-CoV-2 antibodies
Surface plasmon resonance of SARS-Cov-2 antibodies binding to immobilized SARS-Cov-2 RBD was carried out with a Biacore T200 instrument.
Panel A. Kinetic parameters for CR3022 antibodies binding to SARS-Cov-2-RBD determined by SPR. Values are the mean averages from two independent experiments and errors the standard deviation from the mean. Representative SPR sensograms are shown in Figure 1, panels B-E.
Panel B. Kinetic parameters for H4 and B38 antibodies binding to SARS-Cov-2 RBD determined by SPR. Values are the mean averages from two independent experiments and errors the standard deviation from the mean. Representative SPR sensograms are shown in Figure 1, panels F-I.

Supplementary Figure 1

A. CR3022-IgG

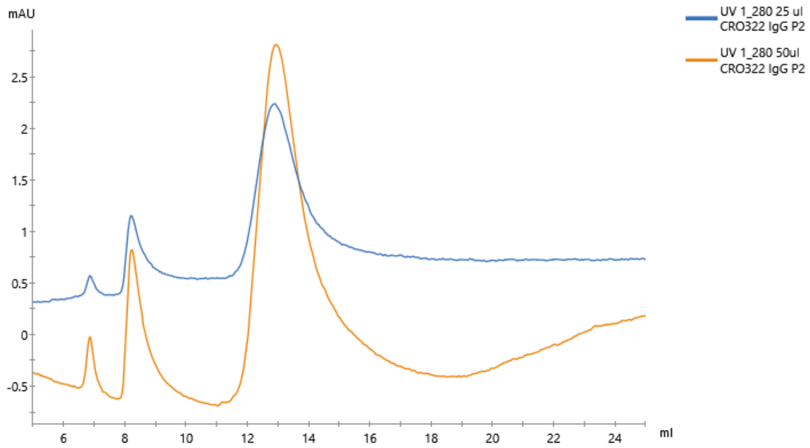

B. CR3022-Fab-TD

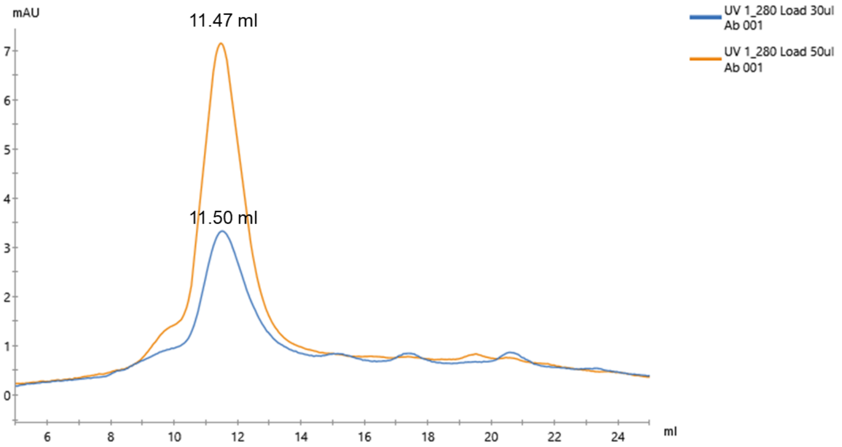

C. CR3022-Fab

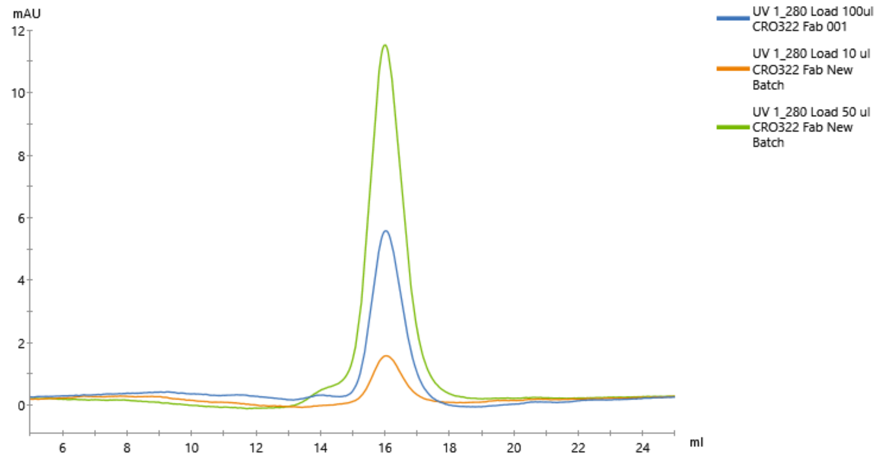

D. Molecular wight standards

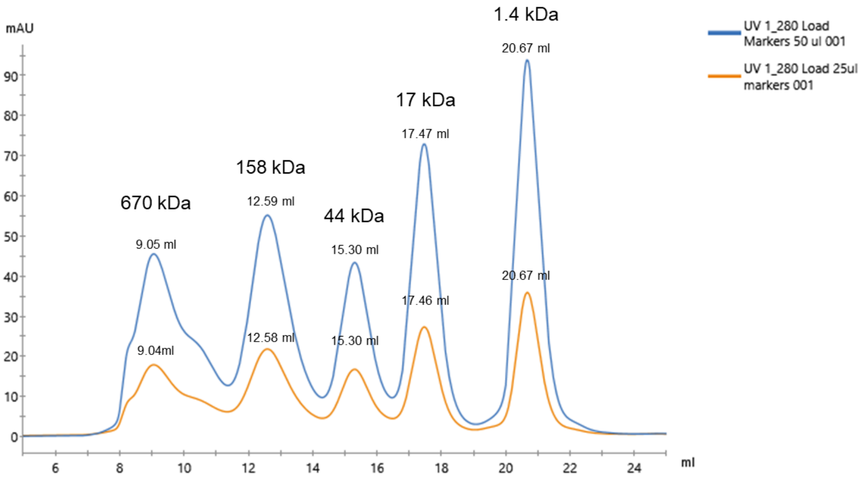

Supplementary Figure 1. Analytical size exclusion chromatography of CR3022 antibodies

The mono-dispersity and molecular weight of CR3022-IgG (panel A, Mw 127.1 kDa), CR3022-Fab-TD (panel B, Mw 252 kDa) and CR3022-Fab (panel C, Mw 32 kDa) were determined by analytical size exclusion chromatography using a Superdex 200 10/300 column. Proteins were injected onto the column using PBS, pH 7.4 at two or three volumes between 10 and 100ul. Chromatograms depict the elution profiles at OD_{280} absorbance. The average molecular weight was calculated by extrapolation from the profiles of Biorad gel filtration standards (panel D).

Supplementary Figure 2

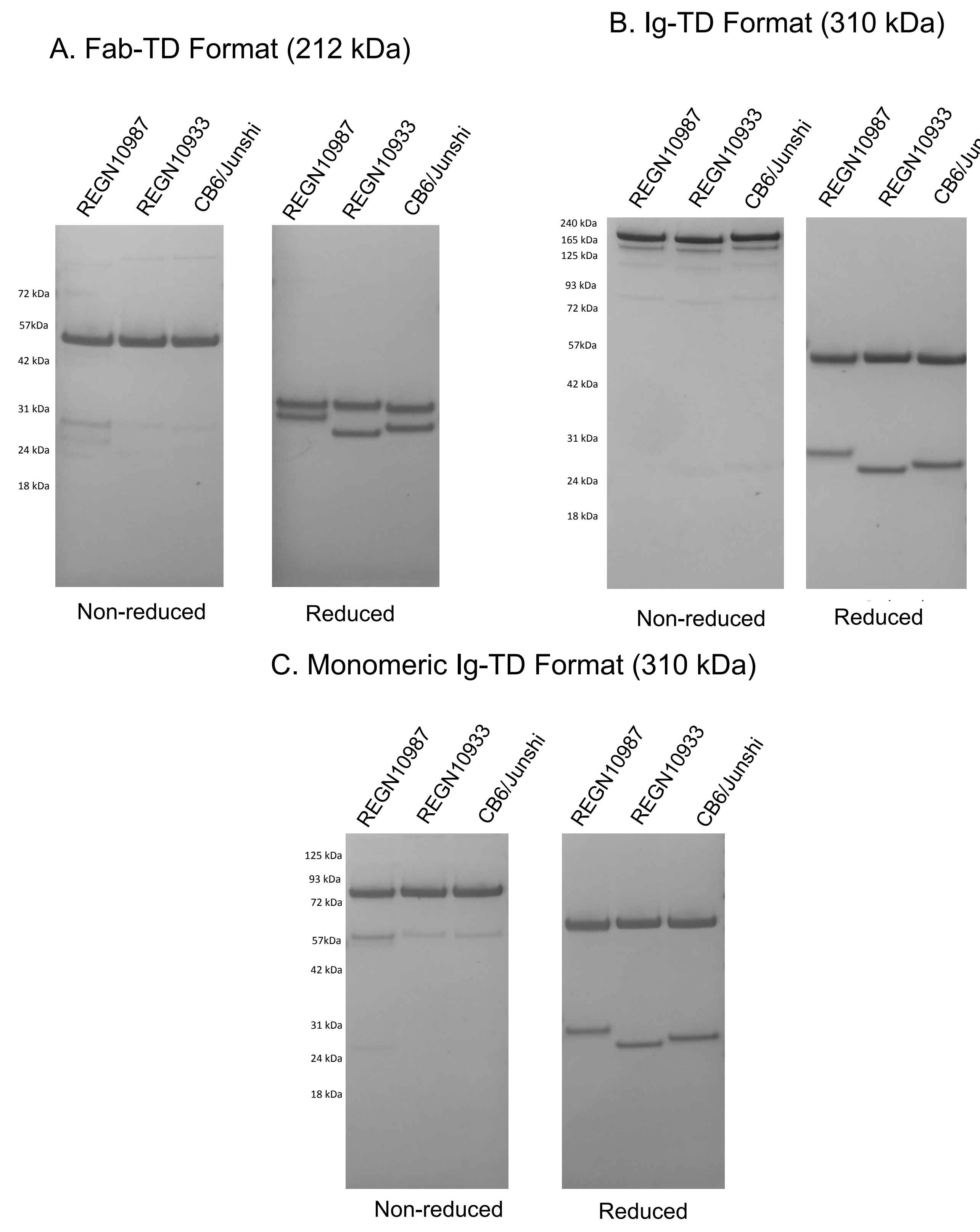

Supplementary Figure 2: SDS-PAGE analysis of reformatted clinical stage SARS-CoV-2 mAbs

The antibodies REGN10987, REGN10933 and CB6/Junshi were formatted into three different multivalent formats (illustrated in supplementary figure 1) and expressed by secretion from Expi293F cells. The proteins were purified using protein A affinity chromatography. The purified proteins were analysed on denaturing SDS-PAGE under non-reduced or under reduced conditions. Panel A. Fab-TD without Fc. Panel B. Ig-TD with fully intact hinge. Panel C. Monomeric Ig-TD devoid of core hinge. Uncropped gels appear in Supplementary figure 8.

Supplementary Figure 3

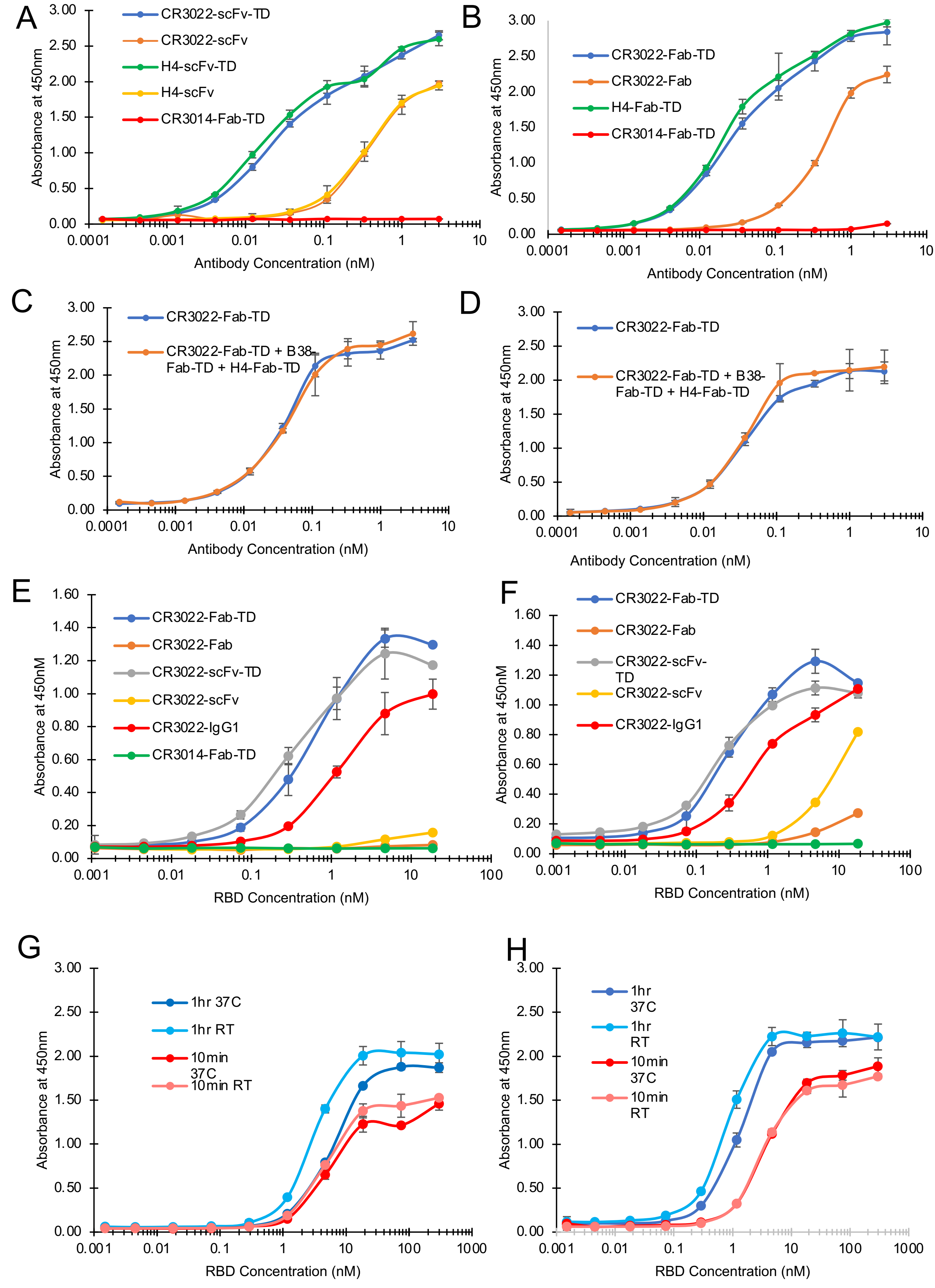

Supplementary Figure 3. ELISA analysis for anti-SARS-CoV-2 antibodies binding to SARS-CoV-2 Spike protein

The potency of binding of various anti-SARS-CoV-2 antibodies was compared in direct ELISAs with either recombinant S1 or RBD protein coated plates. In panels A and B the indicated antibody fragments titrated were scFv or Fab fragments respectively binding to SARS-CoV-2 spike protein S1. The possible additive effects of combining anti-SARS-CoV-2 CR3022-Fab-TD with H4-Fab-TD and B38-Fab-TD was assessed for binding to RBD protein coated plates (panel C) or S1 protein coated plates (D) but there was no advantage observed in the cocktails, even though all three antibodies bind different spike protein epitopes. An additional comparison was carried out with sandwich ELISAs using either ACE2-Fc (panel E) or ACE2-Fc-TD (panel F) coated plates to capture SARS-CoV-2 RBD and detection with the various indicated anti- SARS-CoV-2 antibodies. Further, the detection rates were examined using reduced times of sequential or simultaneous interaction and detection of SARS-CoV-2 RBD captured on immunosorbed ACE2-Fc-TD tetramer. In panel G, ACE2-Fc-TD coated plates captured RBD by incubation for 1 hour or for 10 minutes at either room temperature or 37^0C as shown. After washing, the plates were incubated with 1 nM CR3022-Fab-TD for 1 hour or for 10 minutes, at room temperature or at 37^0C followed by anti-His-HRP antibody incubation for 1 hour or for 10 minutes, also at room temperature or at 37^0C for detection. In panel H, RBD, CR3022-Fab-TD and anti-His-HRP antibody were added simultaneously.

Supplementary Figure 4

A. Wuhan PV

Antibody	PV assay: IC_{50} (pM)	PV assay: fold change compared to IgG
REGN10987 IgG1	59	
REGN10987 Fab-TD	9	7
REGN10987 mIg-TD	5	12
REGN10987 Ig-TD	1	59
REGN10933 IgG1	26	
REGN10933 Fab-TD	12	2
REGN10933 mIg-TD	6	4
REGN10933 Ig-TD	2	13
CB6/Junshi IgG1	249	
CB6/Junshi Fab-TD	20	12
CB6/Junshi mIg-TD	12	21
CB6/Junshi Ig-TD	3	83
H4-scFv-TD	39	

B. Wuhan, Kent and South African PV

	Wuhan PV assay:		B.1.1.7 PV assay:		B.1.351 PV assay:	
Antibody	IC_{50} (pM)	fold change compared to IgG	IC_{50} (pM)	fold change compared to IgG	IC_{50} (pM)	fold change compared to IgG
REGN10987 IgG1	17.6		8.2		7.3	1.0
REGN10987 Fab-TD	6.8	2.6	2.4	3.3	1.1	6.8
REGN10987 mIg-TD	1.4	13.0	0.7	12.6	0.3	21.6
REGN10987 Ig-TD	1.3	13.1	0.5	15.4	0.7	11.1
REGN10933 IgG1	8.7		9.6		N/A	N/A
REGN10933 Fab-TD	3.8	2.3	3.5	2.8	1.0	N/A
REGN10933 mIg-TD	2.4	3.6	1.0	9.2	0.7	N/A
REGN10933 Ig-TD	2.3	3.8	1.0	9.5	1.5	N/A
CB6/Junshi IgG1	50.4		2041.0		N/A	N/A
CB6/Junshi Fab-TD	35.4	1.4	12.8	160.1	N/A	N/A
CB6/Junshi mIg-TD	5.9	8.6	7.0	291.6	N/A	N/A
CB6/Junshi Ig-TD	4.8	10.5	7.5	271.0	N/A	N/A

Supplementary Figure 4. IC50 data for PV neutralization by antibodies.

A. Tabulated picomolar IC50 values of Wuhan PV neutralization by REGN10987, REGN10933 and CB6/Junshi antibodies in the IgG1, Fab-TD, mIg-TD and Ig-TD formats
B. Tabulated picomolar IC50 values of Wuhan, B.1.1.7 and B.1.351 PV neutralization by REGN10987, REGN10933 and CB6/Junshi antibodies in the IgG1, Fab-TD, mIg-TD and Ig-TD formats

Supplementary Figure 5

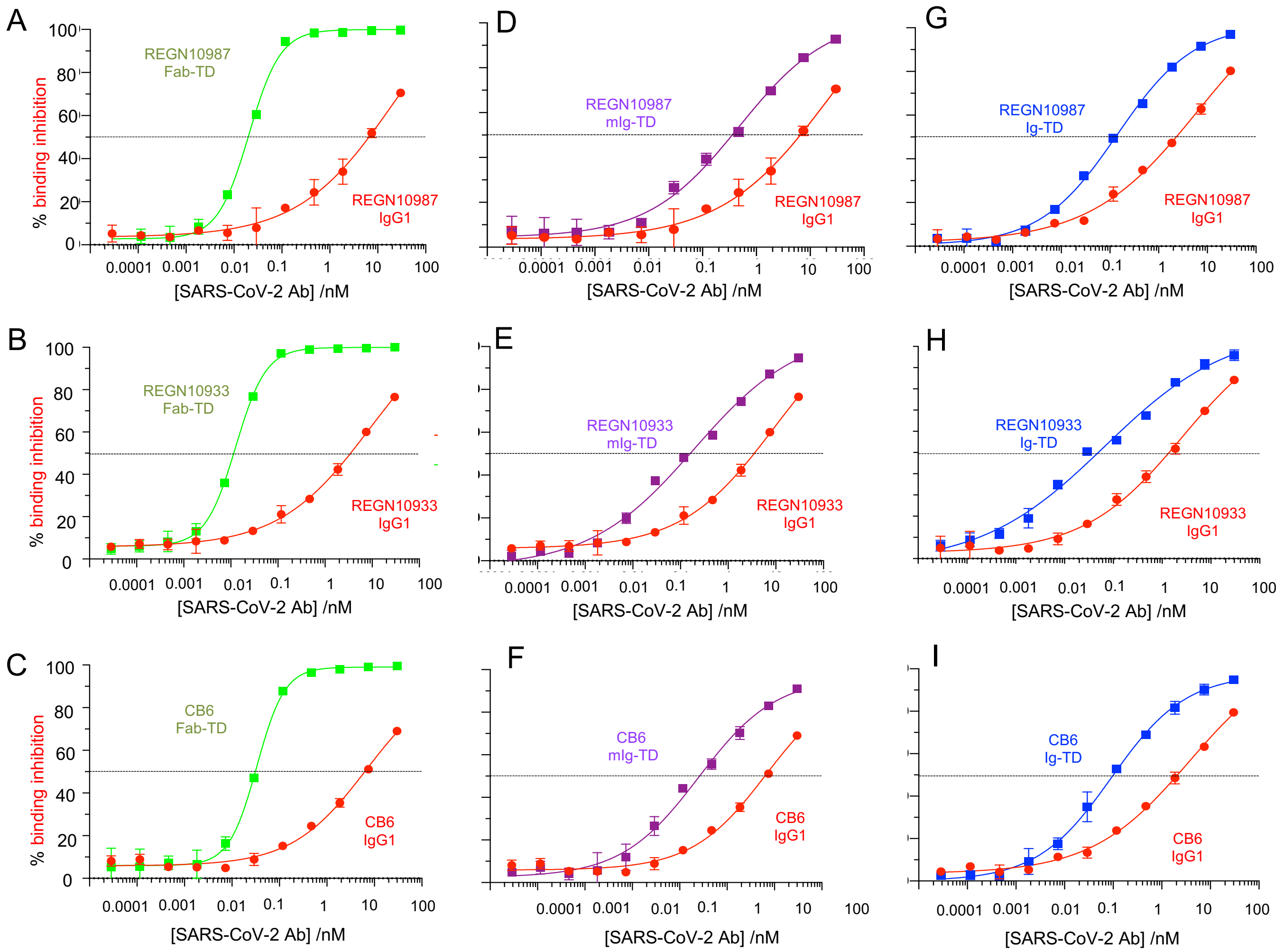

J

Anti-SARS-CoV-2 Antibody	Competitive ELISA			
	IC_{50}		Fold change compared to IgG based on:	
	(nM)	(μg/mL)	IC_{50} (nM)	IC_{50} (μg/mL)
REGN10987 IgG1	7	1.01		
REGN10933 IgG1	3.3	0.47916		
CB6/Junshi IgG1	7	1.02		
REGN10987 Fab-TD	0.02	0.00423	350	239
REGN10933 Fab-TD	0.012	0.00256	275	187
CB6/Junshi Fab-TD	0.03	0.00641	233	159
REGN10987 mIg-TD	0.4	0.12528	18	8
REGN10933 mIg-TD	0.18	0.05677	18	8
CB6/Junshi mIg-TD	0.29	0.09147	24	11

K

Anti-SARS-CoV-2 Antibody	Competitive ELISA			
	IC_{50}		Fold change compared to IgG based on:	
	(nM)	(μg/mL)	IC_{50} (nM)	IC_{50} (μg/mL)
REGN10987 IgG1	2.3	0.33143		
REGN10933 IgG1	1.45	0.21054		
CB6/Junshi IgG1	2	0.2904		
REGN10987 Ig-TD	0.12	0.03696	19	9
REGN10933 Ig-TD	0.045	0.01396	32	15
CB6/Junshi Ig-TD	0.1	0.03102	20	9

Supplementary Figure 5. Competitive ELISA assessing binding inhibition potency enhancement of multivalent mAbs

Competitive ELISA was used to measure percentage binding inhibition of human ACE2-Fc binding to SARS-CoV-2 RBD using three different tetravalent formats of the clinical stage mAbs REGN10987, REGN10933 and CB6/Junshi. The percentage binding inhibition shown in the dose-response profiles for Fab-TD, mIg-TD and Ig-TD formats are presented in panels A-C, D-F, G-I, respectively. For each of the tetravalent mAb formats, the binding inhibitiion potency was compared against the parental mAb in the IgG format and the IC_{50} values, represented by the horizontal dotted lines, were used calculate the fold potency change shown in panels J and K. The experiments were performed in duplicates and the error bars denote standard deviation (n = 2). Panel J shows IC_{50} comparing the Fab-TD and mIg-TD formats with the IgG1 and panel K shows IC_{50} when comparing the Ig-TD formats with the IgG1.

Supplementary Figure 6. Sequences of clinical stage antibodies in tetrameric formats.

The nucleotide and derived amino acid sequence of the clinical stage antibodies engineered into tetrameric quads by incorporating the p53 tetramerization domain. The other antibodies engineered as tetrameric quads are described in (Miller et al., 2021) submitted for publication DOI: 10.21203/rs.3.rs-151560/v1 .

REGN10987 Fab-TD H chain
REGN10987 mIg-TD H chain
REGN10987 Ig-TD H chain
REGN10987 L chain

REGN10933 Fab-TD H chain
REGN10933 mIg-TD H chain
REGN10933 Ig-TD H chain
REGN10933 L chain

CB6 Fab-TD H chain
CB6 mIg-TD H chain
CB6 Ig-TD H chain
CB6 L chain

REGN10987 Fab-TD
CAGGTGCAGCTGGTGGAGTCTGGGGGAGGCGTGGTCCAGCCTGGGAGGTCCCTGAGACTCTC
CTGTGCAGCCTCTGGATTCACCTTCAGTAACTATGCTATGTACTGGGTCCGCCAGGCTCCAG
GCAAGGGGCTGGAGTGGGTGGCAGTTATATCATATGATGGAAGTAATAAATACTATGCAGAC
TCCGTGAAGGGCCGATTCACCATCTCCAGAGACAATTCCAAGAACACGCTGTATCTGCAAAT
GAACAGCCTGAGAACTGAGGACACGGCTGTGTATTACTGTGCGAGTGGCTCCGACTACGGTG
ACTACTTATTGGTTTACTGGGGCCAGGGAACCCTGGTCACCGTCTCCTCAGCCAGCACCAAG
GGCCCCTCTGTGTTCCCTCTGGCCCCTTCCAGCAAGTCCACCTCTGGCGGAACAGCCGCTCT
GGGCTGCCTCGTGAAGGACTACTTCCCCGAGCCTGTGACCGTGTCCTGGAACTCTGGCGCTC
TGACCAGCGGAGTGCACACCTTCCCTGCTGTGCTGCAGTCCTCAGGGCTGTACTCCCTTTCT
AGTGTCGTAACAGTGCCATCTTCTAGCCTGGGGACCCAGACGTACATCTGTAACGTGAATCA
TAAACCCAGTAACACAAAGGTAGATAAGAAGGTTGAACCTAAGTCCTGCGATAAGACACATA
CCAAGAAGAAACCACTGGATGGAGAATATTTCACCCTTCAGATCCGTGGGCGTGAGCGCTTC
GAGATGTTCCGAGAGCTGAATGAGGCCTTGGAACTCAAGGATGCCCAGGCTGGGAAGGAGCC
AGGGCACCACCACCATCACCAC

QVQLVESGGGVVQPGRSLRLSCAASGFTFSNYAMYWVRQAPGKGLEWVAVISYDGSNKYYAD
SVKGRFTISRDNSKNTLYLQMNSLRTEDTAVYYCASGSDYGDYLLVYWGQGTLVTVSSASTK
GPSVFPLAPSSKSTSGGTAALGCLVKDYFPEPVTVSWNSGALTSGVHTFPAVLQSSGLYSLS
SVVTVPSSSLGTQTYICNVNHKPSNTKVDKKVEPKSCDKTHTKKKPLDGEYFTLQIRGRERF
EMFRELNEALELKDAQAGKEPGHHHHHH

REGN10987 mIg-TD
CAGGTGCAGCTGGTGGAGTCTGGGGGAGGCGTGGTCCAGCCTGGGAGGTCCCTGAGACTCTC
CTGTGCAGCCTCTGGATTCACCTTCAGTAACTATGCTATGTACTGGGTCCGCCAGGCTCCAG
GCAAGGGGCTGGAGTGGGTGGCAGTTATATCATATGATGGAAGTAATAAATACTATGCAGAC
TCCGTGAAGGGCCGATTCACCATCTCCAGAGACAATTCCAAGAACACGCTGTATCTGCAAAT
GAACAGCCTGAGAACTGAGGACACGGCTGTGTATTACTGTGCGAGTGGCTCCGACTACGGTG
ACTACTTATTGGTTTACTGGGGCCAGGGAACCCTGGTCACCGTCTCCTCAGCCAGCACCAAG
GGCCCCTCTGTGTTCCCTCTGGCCCCTTCCAGCAAGTCCACCTCTGGCGGAACAGCCGCTCT
GGGCTGCCTCGTGAAGGACTACTTCCCCGAGCCTGTGACCGTGTCCTGGAACTCTGGCGCTC
TGACCAGCGGAGTGCACACCTTCCCTGCTGTGCTGCAGTCCTCAGGGCTGTACTCCCTTTCT
AGTGTCGTAACAGTGCCATCTTCTAGCCTGGGGACCCAGACGTACATCTGTAACGTGAATCA
TAAACCCAGTAACACAAAGGTAGATAAGAAGGTTGAACCTAAGTCCTGCGATAAGACACATA
CCGCCCCTGAACTGCTGGGCGGACCTTCCGTGTTCCTGTTCCCCCCAAAGCCCAAGGACACC

CTGATGATCTCCCGGACCCCCGAAGTGACCTGCGTGGTGGTGGATGTGTCCCACGAGGACCC
TGAAGTGAAGTTCAATTGGTACGTGGACGGCGTGGAAGTGCACAACGCCAAGACCAAGCCTA
GAGAGGAACAGTACAACTCCACCTACCGGGTGGTGTCCGTGCTGACCGTGCTGCACCAGGAT
TGGCTGAACGGCAAAGAGTACAAGTGCAAGGTGTCCAACAAGGCCCTGCCTGCCCCCATCGA
AAAGACCATCTCCAAGGCCAAGGGCCAGCCCCGGGAACCCCAGGTGTACACACTGCCCCCTA
GCAGGGACGAGCTGACCAAGAACCAGGTGTCCCTGACCTGTCTCGTGAAAGGCTTCTACCCC
TCCGATATCGCCGTGGAATGGGAGTCCAACGGCCAGCCTGAGAACAACTACAAGACCACCCC
CCCTGTGCTGGACTCCGACGGCTCATTCTTCCTGTACAGCAAGCTGACAGTGGACAAGTCCC
GGTGGCAGCAGGGCAACGTGTTCTCCTGCTCCGTGATGCACGAGGCCCTGCACAACCACTAC
ACCCAGAAGTCCCTGTCCCTGAGCCCCGGCAAGAAGAAAAAGCCCCTGGACGGCGAGTACTT
CACACTGCAGATCCGGGGCAGAGAACGCTTCGAGATGTTCAGAGAGCTGAACGAGGCCCTGG
AACTGAAGGATGCCCAGGCCGGAAAAGAGCCCGGCGACTACAAGGACGACGACGACAAACAC
CACCATCACCACCAC

QVQLVESGGGVVQPGRSLRLSCAASGFTFSNYAMYWVRQAPGKGLEWVAVISYDGSNKYYAD
SVKGRFTISRDNSKNTLYLQMNSLRTEDTAVYYCASGSDYGDYLLVYWGQGTLVTVSSASTK
GPSVFPLAPSSKSTSGGTAALGCLVKDYFPEPVTVSWNSGALTSGVHTFPAVLQSSGLYSLS
SVVTVPSSSLGTQTYICNVNHKPSNTKVDKKVEPKSCDKTHTAPELLGGPSVFLFPPKPKDT
LMISRTPEVTCVVVDVSHEDPEVKFNWYVDGVEVHNAKTKPREEQYNSTYRVVSVLTVLHQD
WLNGKEYKCKVSNKALPAPIEKTISKAKGQPREPQVYTLPPSRDELTKNQVSLTCLVKGFYP
SDIAVEWESNGQPENNYKTTPPVLDSDGSFFLYSKLTVDKSRWQQGNVFSCSVMHEALHNHY
TQKSLSLSPGKKKKPLDGEYFTLQIRGRERFEMFRELNEALELKDAQAGKEPGDYKDDDDKH
HHHHH

REGN10987 Ig-TD
CAGGTGCAGCTGGTGGAGTCTGGGGGAGGCGTGGTCCAGCCTGGGAGGTCCCTGAGACTCTC
CTGTGCAGCCTCTGGATTCACCTTCAGTAACTATGCTATGTACTGGGTCCGCCAGGCTCCAG
GCAAGGGGCTGGAGTGGGTGGCAGTTATATCATATGATGGAAGTAATAAATACTATGCAGAC
TCCGTGAAGGGCCGATTCACCATCTCCAGAGACAATTCCAAGAACACGCTGTATCTGCAAAT
GAACAGCCTGAGAACTGAGGACACGGCTGTGTATTACTGTGCGAGTGGCTCCGACTACGGTG
ACTACTTATTGGTTTACTGGGGCCAGGGAACCCTGGTCACCGTCTCCTCAGCCAGCACCAAG
GGCCCCTCTGTGTTCCCTCTGGCCCCTTCCAGCAAGTCCACCTCTGGCGGAACAGCCGCTCT
GGGCTGCCTCGTGAAGGACTACTTCCCCGAGCCTGTGACCGTGTCCTGGAACTCTGGCGCTC
TGACCAGCGGAGTGCACACCTTCCCTGCTGTGCTGCAGTCCTCCGGCCTGTACTCCCTGTCC
TCCGTCGTGACCGTGCCTTCCAGCTCTCTGGGCACCCAGACCTACATCTGCAACGTGAACCA
CAAGCCCTCCAACACCAAGGTGGACAAGAAGGTGGAACCCAAGTCCTGCGACAAGACCCACA
CCTGTCCCCCTTGTCCTGCCCCTGAACTGCTGGGCGGACCTTCCGTGTTCCTGTTCCCCCCA
AAGCCCAAGGACACCCTGATGATCTCCCGGACCCCCGAAGTGACCTGCGTGGTGGTGGATGT
GTCCCACGAGGACCCTGAAGTGAAGTTCAATTGGTACGTGGACGGCGTGGAAGTGCACAACG
CCAAGACCAAGCCTAGAGAGGAACAGTACAACAGCACCTACAGAGTGGTGTCCGTGCTGACC
GTGCTGCACCAGGATTGGCTGAACGGCAAAGAGTACAAGTGCAAGGTGTCCAACAAGGCCCT
GCCTGCTCCTATCGAGAAAACCATCAGCAAGGCCAAGGGCCAGCCTAGGGAACCCCAGGTTT
ACACACTGCCTCCAAGCCGGGAAGAGATGACCAAGAACCAGGTGTCCCTGACCTGCCTCGTG
AAGGGCTTCTACCCTTCCGATATCGCCGTGGAATGGGAGAGCAATGGCCAGCCAGAGAACAA
CTACAAGACAACCCCTCCTGTGCTGGACAGCGACGGCTCATTCTTCCTGTACAGCAAGCTGA
CAGTGGACAAGTCCAGATGGCAGCAGGGCAACGTGTTCTCCTGCTCTGTGATGCACGAGGCC
CTGCACAACCACTACACCCAGAAGTCCCTGAGCCTGTCTCCTGGCAAAAAGAAAAAGCCCCT
GGACGGCGAGTACTTCACACTGCAAATCCGGGGCAGAGAACGCTTCGAGATGTTCAGAGAGC
TGAACGAGGCCCTGGAACTGAAGGATGCCCAGGCCGGAAAAGAGCCCGGC

QVQLVESGGGVVQPGRSLRLSCAASGFTFSNYAMYWVRQAPGKGLEWVAVISYDGSNKYYAD
SVKGRFTISRDNSKNTLYLQMNSLRTEDTAVYYCASGSDYGDYLLVYWGQGTLVTVSSASTK
GPSVFPLAPSSKSTSGGTAALGCLVKDYFPEPVTVSWNSGALTSGVHTFPAVLQSSGLYSLS
SVVTVPSSSLGTQTYICNVNHKPSNTKVDKKVEPKSCDKTHTCPPCPAPELLGGPSVFLFPP
KPKDTLMISRTPEVTCVVVDVSHEDPEVKFNWYVDGVEVHNAKTKPREEQYNSTYRVVSVLT
VLHQDWLNGKEYKCKVSNKALPAPIEKTISKAKGQPREPQVYTLPPSREEMTKNQVSLTCLV

KGFYPSDIAVEWESNGQPENNYKTTPPVLDSDGSFFLYSKLTVDKSRWQQGNVFSCSVMHEA
LHNHYTQKSLSLSPGKKKKPLDGEYFTLQIRGRERFEMFRELNEALELKDAQAGKEPG

REGN10987 LC
CAGTCTGCCCTGACTCAGCCTGCCTCCGTGTCTGGGTCTCCTGGACAGTCGATCACCATCTC
CTGCACTGGAACCAGCAGTGACGTTGGTGGTTATAACTATGTCTCCTGGTACCAACAACACC
CAGGCAAAGCCCCCAAACTCATGATTTATGATGTCAGTAAGCGGCCCTCAGGGGTTTCTAAT
CGCTTCTCTGGCTCCAAGTCTGGCAACACGGCCTCCCTGACCATCTCTGGGCTCCAGTCTGA
GGACGAGGCTGATTATTACTGCAACTCTTTGACAAGCATCAGCACTTGGGTGTTCGGCGGAG
GGACCAAGCTGACCGTCCTAGGACAGCCAAAAGCAGCCCCATCCGTAACTCTGTTCCCACCT
AGTTCAGAGGAGCTTCAAGCAAACAAAGCCACACTTGTTTGCCTTATTAGTGATTTTTATCC
CGGTGCCGTGACAGTTGCCTGGAAAGCTGATAGCTCACCAGTGAAAGCTGGCGTGGAGACAA
CCACACCATCTAAACAAAGCAATAACAAGTATGCTGCCAGCTCATATCTGAGTCTCACTCCA
GAACAATGGAAGTCTCATCGGTCCTATAGCTGTCAAGTGACCCACGAAGGCAGTACCGTCGA
GAAGACCGTGGCACCAACAGAGTGTAGC

QSALTQPASVSGSPGQSITISCTGTSSDVGGYNYVSWYQQHPGKAPKLMIYDVSKRPSGVSN
RFSGSKSGNTASLTISGLQSEDEADYYCNSLTSISTWVFGGGTKLTVLGQPKAAPSVTLFPP
SSEELQANKATLVCLISDFYPGAVTVAWKADSSPVKAGVETTTPSKQSNNKYAASSYLSLTP
EQWKSHRSYSCQVTHEGSTVEKTVAPTECS

REGN10933 Fab-TD
CAGGTGCAGCTGGTGGAGTCTGGGGGAGGCTTGGTCAAGCCTGGAGGGTCCCTGAGACTCTC
CTGTGCAGCCTCTGGATTCACCTTCAGTGACTACTACATGAGCTGGATCCGCCAGGCTCCAG
GGAAGGGGCTGGAGTGGGTTTCATACATTACTTATAGTGGTAGTACCATATACTACGCAGAC
TCTGTGAAGGGCCGATTCACCATCTCCAGGGACAACGCCAAGAGCTCACTGTATCTGCAAAT
GAACAGCCTGAGAGCCGAGGACACGGCCGTGTATTACTGTGCGAGAGATCGCGGTACAACTA
TGGTCCCCTTTGACTACTGGGGCCAGGGAACCCTGGTCACCGTCTCCTCAGCCAGCACCAAG
GGCCCCTCTGTGTTCCCTCTGGCCCCTTCCAGCAAGTCCACCTCTGGCGGAACAGCCGCTCT
GGGCTGCCTCGTGAAGGACTACTTCCCCGAGCCTGTGACCGTGTCCTGGAACTCTGGCGCTC
TGACCAGCGGAGTGCACACCTTCCCTGCTGTGCTGCAGTCCTCAGGGCTGTACTCCCTTTCT
AGTGTCGTAACAGTGCCATCTTCTAGCCTGGGGACCCAGACGTACATCTGTAACGTGAATCA
TAAACCCAGTAACACAAAGGTAGATAAGAAGGTTGAACCTAAGTCCTGCGATAAGACACATA
CCAAGAAGAAACCACTGGATGGAGAATATTTCACCCTTCAGATCCGTGGGCGTGAGCGCTTC
GAGATGTTCCGAGAGCTGAATGAGGCCTTGGAACTCAAGGATGCCCAGGCTGGGAAGGAGCC
AGGGCACCACCACCATCACCAC

QVQLVESGGGLVKPGGSLRLSCAASGFTFSDYYMSWIRQAPGKGLEWVSYITYSGSTIYYAD
SVKGRFTISRDNAKSSLYLQMNSLRAEDTAVYYCARDRGTTMVPFDYWGQGTLVTVSSASTK
GPSVFPLAPSSKSTSGGTAALGCLVKDYFPEPVTVSWNSGALTSGVHTFPAVLQSSGLYSLS
SVVTVPSSSLGTQTYICNVNHKPSNTKVDKKVEPKSCDKTHTKKKPLDGEYFTLQIRGRERF
EMFRELNEALELKDAQAGKEPGHHHHHH

REGN10933 mIg-TD
CAGGTGCAGCTGGTGGAGTCTGGGGGAGGCTTGGTCAAGCCTGGAGGGTCCCTGAGACTCTC
CTGTGCAGCCTCTGGATTCACCTTCAGTGACTACTACATGAGCTGGATCCGCCAGGCTCCAG
GGAAGGGGCTGGAGTGGGTTTCATACATTACTTATAGTGGTAGTACCATATACTACGCAGAC
TCTGTGAAGGGCCGATTCACCATCTCCAGGGACAACGCCAAGAGCTCACTGTATCTGCAAAT
GAACAGCCTGAGAGCCGAGGACACGGCCGTGTATTACTGTGCGAGAGATCGCGGTACAACTA
TGGTCCCCTTTGACTACTGGGGCCAGGGAACCCTGGTCACCGTCTCCTCAGCCAGCACCAAG
GGCCCCTCTGTGTTCCCTCTGGCCCCTTCCAGCAAGTCCACCTCTGGCGGAACAGCCGCTCT
GGGCTGCCTCGTGAAGGACTACTTCCCCGAGCCTGTGACCGTGTCCTGGAACTCTGGCGCTC
TGACCAGCGGAGTGCACACCTTCCCTGCTGTGCTGCAGTCCTCAGGGCTGTACTCCCTTTCT
AGTGTCGTAACAGTGCCATCTTCTAGCCTGGGGACCCAGACGTACATCTGTAACGTGAATCA
TAAACCCAGTAACACAAAGGTAGATAAGAAGGTTGAACCTAAGTCCTGCGATAAGACACATA
CCGCCCCTGAACTGCTGGGCGGACCTTCCGTGTTCCTGTTCCCCCCAAAGCCCAAGGACACC
CTGATGATCTCCCGGACCCCCGAAGTGACCTGCGTGGTGGTGGATGTGTCCCACGAGGACCC

TGAAGTGAAGTTCAATTGGTACGTGGACGGCGTGGAAGTGCACAACGCCAAGACCAAGCCTA
GAGAGGAACAGTACAACTCCACCTACCGGGTGGTGTCCGTGCTGACCGTGCTGCACCAGGAT
TGGCTGAACGGCAAAGAGTACAAGTGCAAGGTGTCCAACAAGGCCCTGCCTGCCCCCATCGA
AAAGACCATCTCCAAGGCCAAGGGCCAGCCCCGGGAACCCCAGGTGTACACACTGCCCCCTA
GCAGGGACGAGCTGACCAAGAACCAGGTGTCCCTGACCTGTCTCGTGAAAGGCTTCTACCCC
TCCGATATCGCCGTGGAATGGGAGTCCAACGGCCAGCCTGAGAACAACTACAAGACCACCCC
CCCTGTGCTGGACTCCGACGGCTCATTCTTCCTGTACAGCAAGCTGACAGTGGACAAGTCCC
GGTGGCAGCAGGGCAACGTGTTCTCCTGCTCCGTGATGCACGAGGCCCTGCACAACCACTAC
ACCCAGAAGTCCCTGTCCCTGAGCCCCGGCAAGAAGAAAAAGCCCCTGGACGGCGAGTACTT
CACACTGCAGATCCGGGGCAGAGAACGCTTCGAGATGTTCAGAGAGCTGAACGAGGCCCTGG
AACTGAAGGATGCCCAGGCCGGAAAAGAGCCCGGCGACTACAAGGACGACGACGACAAACAC
CACCATCACCACCAC

QVQLVESGGGLVKPGGSLRLSCAASGFTFSDYYMSWIRQAPGKGLEWVSYITYSGSTIYYAD
SVKGRFTISRDNAKSSLYLQMNSLRAEDTAVYYCARDRGTTMVPFDYWGQGTLVTVSSASTK
GPSVFPLAPSSKSTSGGTAALGCLVKDYFPEPVTVSWNSGALTSGVHTFPAVLQSSGLYSLS
SVVTVPSSSLGTQTYICNVNHKPSNTKVDKKVEPKSCDKTHTAPELLGGPSVFLFPPKPKDT
LMISRTPEVTCVVVDVSHEDPEVKFNWYVDGVEVHNAKTKPREEQYNSTYRVVSVLTVLHQD
WLNGKEYKCKVSNKALPAPIEKTISKAKGQPREPQVYTLPPSRDELTKNQVSLTCLVKGFYP
SDIAVEWESNGQPENNYKTTPPVLDSDGSFFLYSKLTVDKSRWQQGNVFSCSVMHEALHNHY
TQKSLSLSPGKKKKPLDGEYFTLQIRGRERFEMFRELNEALELKDAQAGKEPGDYKDDDDKH
HHHHH

REGN10933 Ig-TD
CAGGTGCAGCTGGTGGAGTCTGGGGGAGGCTTGGTCAAGCCTGGAGGGTCCCTGAGACTCTC
CTGTGCAGCCTCTGGATTCACCTTCAGTGACTACTACATGAGCTGGATCCGCCAGGCTCCAG
GGAAGGGGCTGGAGTGGGTTTCATACATTACTTATAGTGGTAGTACCATATACTACGCAGAC
TCTGTGAAGGGCCGATTCACCATCTCCAGGGACAACGCCAAGAGCTCACTGTATCTGCAAAT
GAACAGCCTGAGAGCCGAGGACACGGCCGTGTATTACTGTGCGAGAGATCGCGGTACAACTA
TGGTCCCCTTTGACTACTGGGGCCAGGGAACCCTGGTCACCGTCTCCTCAGCCAGCACCAAG
GGCCCCTCTGTGTTCCCTCTGGCCCCTTCCAGCAAGTCCACCTCTGGCGGAACAGCCGCTCT
GGGCTGCCTCGTGAAGGACTACTTCCCCGAGCCTGTGACCGTGTCCTGGAACTCTGGCGCTC
TGACCAGCGGAGTGCACACCTTCCCTGCTGTGCTGCAGTCCTCCGGCCTGTACTCCCTGTCC
TCCGTCGTGACCGTGCCTTCCAGCTCTCTGGGCACCCAGACCTACATCTGCAACGTGAACCA
CAAGCCCTCCAACACCAAGGTGGACAAGAAGGTGGAACCCAAGTCCTGCGACAAGACCCACA
CCTGTCCCCCTTGTCCTGCCCCTGAACTGCTGGGCGGACCTTCCGTGTTCCTGTTCCCCCCA
AAGCCCAAGGACACCCTGATGATCTCCCGGACCCCCGAAGTGACCTGCGTGGTGGTGGATGT
GTCCCACGAGGACCCTGAAGTGAAGTTCAATTGGTACGTGGACGGCGTGGAAGTGCACAACG
CCAAGACCAAGCCTAGAGAGGAACAGTACAACAGCACCTACAGAGTGGTGTCCGTGCTGACC
GTGCTGCACCAGGATTGGCTGAACGGCAAAGAGTACAAGTGCAAGGTGTCCAACAAGGCCCT
GCCTGCTCCTATCGAGAAAACCATCAGCAAGGCCAAGGGCCAGCCTAGGGAACCCCAGGTTT
ACACACTGCCTCCAAGCCGGGAAGAGATGACCAAGAACCAGGTGTCCCTGACCTGCCTCGTG
AAGGGCTTCTACCCTTCCGATATCGCCGTGGAATGGGAGAGCAATGGCCAGCCAGAGAACAA
CTACAAGACAACCCCTCCTGTGCTGGACAGCGACGGCTCATTCTTCCTGTACAGCAAGCTGA
CAGTGGACAAGTCCAGATGGCAGCAGGGCAACGTGTTCTCCTGCTCTGTGATGCACGAGGCC
CTGCACAACCACTACACCCAGAAGTCCCTGAGCCTGTCTCCTGGCAAAAAGAAAAAGCCCCT
GGACGGCGAGTACTTCACACTGCAAATCCGGGGCAGAGAACGCTTCGAGATGTTCAGAGAGC
TGAACGAGGCCCTGGAACTGAAGGATGCCCAGGCCGGAAAAGAGCCCGGC

QVQLVESGGGLVKPGGSLRLSCAASGFTFSDYYMSWIRQAPGKGLEWVSYITYSGSTIYYAD
SVKGRFTISRDNAKSSLYLQMNSLRAEDTAVYYCARDRGTTMVPFDYWGQGTLVTVSSASTK
GPSVFPLAPSSKSTSGGTAALGCLVKDYFPEPVTVSWNSGALTSGVHTFPAVLQSSGLYSLS
SVVTVPSSSLGTQTYICNVNHKPSNTKVDKKVEPKSCDKTHTCPPCPAPELLGGPSVFLFPP
KPKDTLMISRTPEVTCVVVDVSHEDPEVKFNWYVDGVEVHNAKTKPREEQYNSTYRVVSVLT
VLHQDWLNGKEYKCKVSNKALPAPIEKTISKAKGQPREPQVYTLPPSREEMTKNQVSLTCLV
KGFYPSDIAVEWESNGQPENNYKTTPPVLDSDGSFFLYSKLTVDKSRWQQGNVFSCSVMHEA
LHNHYTQKSLSLSPGKKKKPLDGEYFTLQIRGRERFEMFRELNEALELKDAQAGKEPG

REGN10933 LC
GACATCCAGATGACCCAGTCTCCATCCTCCCTGTCTGCATCTGTAGGAGACAGAGTCACCAT
CACTTGCCAGGCGAGTCAGGACATTACCAACTATTTAAATTGGTATCAGCAGAAACCAGGGA
AAGCCCCTAAGCTCCTGATCTACGCTGCATCCAATTTGGAAACAGGGGTCCCATCAAGGTTC
AGTGGAAGTGGATCTGGGACAGATTTTACTTTCACCATCAGCGGCCTGCAGCCTGAAGATAT
TGCAACATATTACTGTCAACAGTATGATAATCTCCCTCTCACTTTCGGCGGAGGGACCAAGG
TGGAGATCAAACGTACGGTGGCCGCTCCCTCCGTGTTCATCTTCCCACCTTCCGACGAGCAG
CTGAAGTCCGGCACCGCTTCTGTCGTGTGCCTGCTGAACAACTTCTACCCCCGCGAGGCCAA
GGTGCAGTGGAAGGTGGACAACGCCCTGCAGTCCGGCAACTCCCAGGAATCCGTGACCGAGC
AGGACTCCAAGGACAGCACCTACTCCCTGTCCTCCACCCTGACCCTGTCCAAGGCCGACTAC
GAGAAGCACAAGGTGTACGCCTGCGAAGTGACCCACCAGGGCCTGTCTAGCCCCGTGACCAA
GTCTTTCAACCGGGGCGAGTGT

DIQMTQSPSSLSASVGDRVTITCQASQDITNYLNWYQQKPGKAPKLLIYAASNLETGVPSRF
SGSGSGTDFTFTISGLQPEDIATYYCQQYDNLPLTFGGGTKVEIKRTVAAPSVFIFPPSDEQ
LKSGTASVVCLLNNFYPREAKVQWKVDNALQSGNSQESVTEQDSKDSTYSLSSTLTLSKADY
EKHKVYACEVTHQGLSSPVTKSFNRGEC

CB6 Fab-TD
GAGGTGCAGCTGGTTGAATCTGGCGGAGGACTGGTTCAGCCTGGCGGATCTCTGAGACTGTC
TTGTGCCGCCAGCGGCTTTACCGTGTCCAGCAACTACATGAGCTGGGTCCGACAGGCCCCTG
GCAAAGGACTTGAATGGGTGTCCGTGATCTACAGCGGCGGCAGCACCTTTTACGCCGACTCT
GTGAAGGGCAGATTCACCATCAGCCGGGACAACTCTATGAATACCCTGTTCCTTCAGATGAA
CAGCCTGAGAGCCGAGGACACCGCCGTGTACTATTGTGCCAGAGTGCTGCCTATGTACGGCG
ACTACCTGGACTATTGGGGCCAGGGCACACTGGTCACAGTGTCTAGTGCCAGCACCAAGGGC
CCCTCTGTGTTCCCTCTGGCCCCTTCCAGCAAGTCCACCTCTGGCGGAACAGCCGCTCTGGG
CTGCCTCGTGAAGGACTACTTCCCCGAGCCTGTGACCGTGTCCTGGAACTCTGGCGCTCTGA
CCAGCGGAGTGCACACCTTCCCTGCTGTGCTGCAGTCCTCAGGGCTGTACTCCCTTTCTAGT
GTCGTAACAGTGCCATCTTCTAGCCTGGGGACCCAGACGTACATCTGTAACGTGAATCATAA
ACCCAGTAACACAAAGGTAGATAAGAAGGTTGAACCTAAGTCCTGCGATAAGACACATACCA
AGAAGAAACCACTGGATGGAGAATATTTCACCCTTCAGATCCGTGGGCGTGAGCGCTTCGAG
ATGTTCCGAGAGCTGAATGAGGCCTTGGAACTCAAGGATGCCCAGGCTGGGAAGGAGCCAGG
GCACCACCACCATCACCAC

EVQLVESGGGLVQPGGSLRLSCAASGFTVSSNYMSWVRQAPGKGLEWVSVIYSGGSTFYADS
VKGRFTISRDNSMNTLFLQMNSLRAEDTAVYYCARVLPMYGDYLDYWGQGTLVTVSSASTKG
PSVFPLAPSSKSTSGGTAALGCLVKDYFPEPVTVSWNSGALTSGVHTFPAVLQSSGLYSLSS
VVTVPSSSLGTQTYICNVNHKPSNTKVDKKVEPKSCDKTHTKKKPLDGEYFTLQIRGRERFE
MFRELNEALELKDAQAGKEPGHHHHHH

CB6 mIg-TD
GAGGTGCAGCTGGTTGAATCTGGCGGAGGACTGGTTCAGCCTGGCGGATCTCTGAGACTGTC
TTGTGCCGCCAGCGGCTTTACCGTGTCCAGCAACTACATGAGCTGGGTCCGACAGGCCCCTG
GCAAAGGACTTGAATGGGTGTCCGTGATCTACAGCGGCGGCAGCACCTTTTACGCCGACTCT
GTGAAGGGCAGATTCACCATCAGCCGGGACAACTCTATGAATACCCTGTTCCTtCAGATGAA
CAGCCTGAGAGCCGAGGACACCGCCGTGTACTATTGTGCCAGAGTGCTGCCTATGTACGGCG
ACTACCTGGACTATTGGGGCCAGGGCACACTGGTCACAGTGTCTAGTGCCAGCACCAAGGGC
CCCTCTGTGTTCCCTCTGGCCCCTTCCAGCAAGTCCACCTCTGGCGGAACAGCCGCTCTGGG
CTGCCTCGTGAAGGACTACTTCCCCGAGCCTGTGACCGTGTCCTGGAACTCTGGCGCTCTGA
CCAGCGGAGTGCACACCTTCCCTGCTGTGCTGCAGTCCTCAGGGCTGTACTCCCTTTCTAGT
GTCGTAACAGTGCCATCTTCTAGCCTGGGGACCCAGACGTACATCTGTAACGTGAATCATAA
ACCCAGTAACACAAAGGTAGATAAGAAGGTTGAACCTAAGTCCTGCGATAAGACACATACCG
CCCCTGAACTGCTGGGCGGACCTTCCGTGTTCCTGTTCCCCCCAAAGCCCAAGGACACCCTG
ATGATCTCCCGGACCCCCGAAGTGACCTGCGTGGTGGTGGATGTGTCCCACGAGGACCCTGA
AGTGAAGTTCAATTGGTACGTGGACGGCGTGGAAGTGCACAACGCCAAGACCAAGCCTAGAG
AGGAACAGTACAACTCCACCTACCGGGTGGTGTCCGTGCTGACCGTGCTGCACCAGGATTGG

CTGAACGGCAAAGAGTACAAGTGCAAGGTGTCCAACAAGGCCCTGCCTGCCCCCATCGAAAA
GACCATCTCCAAGGCCAAGGGCCAGCCCCGGGAACCCCAGGTGTACACACTGCCCCCTAGCA
GGGACGAGCTGACCAAGAACCAGGTGTCCCTGACCTGTCTCGTGAAAGGCTTCTACCCCTCC
GATATCGCCGTGGAATGGGAGTCCAACGGCCAGCCTGAGAACAACTACAAGACCACCCCCCC
TGTGCTGGACTCCGACGGCTCATTCTTCCTGTACAGCAAGCTGACAGTGGACAAGTCCCGGT
GGCAGCAGGGCAACGTGTTCTCCTGCTCCGTGATGCACGAGGCCCTGCACAACCACTACACC
CAGAAGTCCCTGTCCCTGAGCCCCGGCAAGAAGAAAAAGCCCCTGGACGGCGAGTACTTCAC
ACTGCAGATCCGGGGCAGAGAACGCTTCGAGATGTTCAGAGAGCTGAACGAGGCCCTGGAAC
TGAAGGATGCCCAGGCCGGAAAAGAGCCCGGCGACTACAAGGACGACGACGACAAACACCAC
CATCACCACCAC

EVQLVESGGGLVQPGGSLRLSCAASGFTVSSNYMSWVRQAPGKGLEWVSVIYSGGSTFYADS
VKGRFTISRDNSMNTLFLQMNSLRAEDTAVYYCARVLPMYGDYLDYWGQGTLVTVSSASTKG
PSVFPLAPSSKSTSGGTAALGCLVKDYFPEPVTVSWNSGALTSGVHTFPAVLQSSGLYSLSS
VVTVPSSSLGTQTYICNVNHKPSNTKVDKKVEPKSCDKTHTAPELLGGPSVFLFPPKPKDTL
MISRTPEVTCVVVDVSHEDPEVKFNWYVDGVEVHNAKTKPREEQYNSTYRVVSVLTVLHQDW
LNGKEYKCKVSNKALPAPIEKTISKAKGQPREPQVYTLPPSRDELTKNQVSLTCLVKGFYPS
DIAVEWESNGQPENNYKTTPPVLDSDGSFFLYSKLTVDKSRWQQGNVFSCSVMHEALHNHYT
QKSLSLSPGKKKKPLDGEYFTLQIRGRERFEMFRELNEALELKDAQAGKEPGDYKDDDDKHH
HHHH

CB6 Ig-TD
GAGGTGCAGCTGGTTGAATCTGGCGGAGGACTGGTTCAGCCTGGCGGATCTCTGAGACTGTC
TTGTGCCGCCAGCGGCTTTACCGTGTCCAGCAACTACATGAGCTGGGTCCGACAGGCCCCTG
GCAAAGGACTTGAATGGGTGTCCGTGATCTACAGCGGCGGCAGCACCTTTTACGCCGACTCT
GTGAAGGGCAGATTCACCATCAGCCGGGACAACTCTATGAATACCCTGTTCCTtCAGATGAA
CAGCCTGAGAGCCGAGGACACCGCCGTGTACTATTGTGCCAGAGTGCTGCCTATGTACGGCG
ACTACCTGGACTATTGGGGCCAGGGCACACTGGTCACAGTGTCTAGTGCCAGCACCAAGGGC
CCCTCTGTGTTCCCTCTGGCCCCTTCCAGCAAGTCCACCTCTGGCGGAACAGCCGCTCTGGG
CTGCCTCGTGAAGGACTACTTCCCCGAGCCTGTGACCGTGTCCTGGAACTCTGGCGCTCTGA
CCAGCGGAGTGCACACCTTCCCTGCTGTGCTGCAGTCCTCCGGCCTGTACTCCCTGTCCTCC
GTCGTGACCGTGCCTTCCAGCTCTCTGGGCACCCAGACCTACATCTGCAACGTGAACCACAA
GCCCTCCAACACCAAGGTGGACAAGAAGGTGGAACCCAAGTCCTGCGACAAGACCCACACCT
GTCCCCCTTGTCCTGCCCCTGAACTGCTGGGCGGACCTTCCGTGTTCCTGTTCCCCCCAAAG
CCCAAGGACACCCTGATGATCTCCCGGACCCCCGAAGTGACCTGCGTGGTGGTGGATGTGTC
CCACGAGGACCCTGAAGTGAAGTTCAATTGGTACGTGGACGGCGTGGAAGTGCACAACGCCA
AGACCAAGCCTAGAGAGGAACAGTACAACAGCACCTACAGAGTGGTGTCCGTGCTGACCGTG
CTGCACCAGGATTGGCTGAACGGCAAAGAGTACAAGTGCAAGGTGTCCAACAAGGCCCTGCC
TGCTCCTATCGAGAAAACCATCAGCAAGGCCAAGGGCCAGCCTAGGGAACCCCAGGTTTACA
CACTGCCTCCAAGCCGGGAAGAGATGACCAAGAACCAGGTGTCCCTGACCTGCCTCGTGAAG
GGCTTCTACCCTTCCGATATCGCCGTGGAATGGGAGAGCAATGGCCAGCCAGAGAACAACTA
CAAGACAACCCCTCCTGTGCTGGACAGCGACGGCTCATTCTTCCTGTACAGCAAGCTGACAG
TGGACAAGTCCAGATGGCAGCAGGGCAACGTGTTCTCCTGCTCTGTGATGCACGAGGCCCTG
CACAACCACTACACCCAGAAGTCCCTGAGCCTGTCTCCTGGCAAAAAGAAAAAGCCCCTGGA
CGGCGAGTACTTCACACTGCAAATCCGGGGCAGAGAACGCTTCGAGATGTTCAGAGAGCTGA
ACGAGGCCCTGGAACTGAAGGATGCCCAGGCCGGAAAAGAGCCCGGC

EVQLVESGGGLVQPGGSLRLSCAASGFTVSSNYMSWVRQAPGKGLEWVSVIYSGGSTFYADS
VKGRFTISRDNSMNTLFLQMNSLRAEDTAVYYCARVLPMYGDYLDYWGQGTLVTVSSASTKG
PSVFPLAPSSKSTSGGTAALGCLVKDYFPEPVTVSWNSGALTSGVHTFPAVLQSSGLYSLSS
VVTVPSSSLGTQTYICNVNHKPSNTKVDKKVEPKSCDKTHTCPPCPAPELLGGPSVFLFPPK
PKDTLMISRTPEVTCVVVDVSHEDPEVKFNWYVDGVEVHNAKTKPREEQYNSTYRVVSVLTV
LHQDWLNGKEYKCKVSNKALPAPIEKTISKAKGQPREPQVYTLPPSREEMTKNQVSLTCLVK
GFYPSDIAVEWESNGQPENNYKTTPPVLDSDGSFFLYSKLTVDKSRWQQGNVFSCSVMHEAL
HNHYTQKSLSLSPGKKKKPLDGEYFTLQIRGRERFEMFRELNEALELKDAQAGKEPG

CB6 LC

```
GACATCGTGATGACACAGAGCCCTAGCAGCCTGTCTGCCAGCGTGGGAGACAGAGTGACCAT
CACCTGTAGAGCCAGCCAGAGCATCAGCAGATACCTGAACTGGTATCAGCAGAAGCCCGGCA
AGGCCCCTAAGCTGCTGATCTATGCTGCCAGCTCTCTGCAGTCTGGCGTGCCCTCTAGATTT
TCTGGCAGCGGCTCTGGCACCGACTTCACCCTGACCATATCTAGCCTGCAGCCTGAGGACTT
CGCCACCTACTACTGCCAGCAGAGCTACAGCACCCCTCCTGAGTACACATTTGGCCAGGGCA
CCAAGCTGGAAATCAAGCGTACGGTGGCCGCTCCCTCCGTGTTCATCTTCCCACCTTCCGAC
GAGCAGCTGAAGTCCGGCACCGCTTCTGTCGTGTGCCTGCTGAACAACTTCTACCCCCGCGA
GGCCAAGGTGCAGTGGAAGGTGGACAACGCCCTGCAGTCCGGCAACTCCCAGGAATCCGTGA
CCGAGCAGGACTCCAAGGACAGCACCTACTCCCTGTCCTCCACCCTGACCCTGTCCAAGGCC
GACTACGAGAAGCACAAGGTGTACGCCTGCGAAGTGACCCACCAGGGCCTGTCTAGCCCCGT
GACCAAGTCTTTCAACCGGGGCGAGTGT
```

```
DIVMTQSPSSLSASVGDRVTITCRASQSISRYLNWYQQKPGKAPKLLIYAASSLQSGVPSRF
SGSGSGTDFTLTISSLQPEDFATYYCQQSYSTPPEYTFGQGTKLEIKRTVAAPSVFIFPPSD
EQLKSGTASVVCLLNNFYPREAKVQWKVDNALQSGNSQESVTEQDSKDSTYSLSSTLTLSKA
DYEKHKVYACEVTHQGLSSPVTKSFNRGEC
```


Miller, A., Leach, A., Thomas, J., McAndrew, C., Bentley, E., Mattiuzzo, G., John, L., Mirazimi, A., Harris, G., Gamage, N., *et al.* (2021). A super-potent tetramerized ACE2 protein displays enhanced neutralization of SARS-CoV-2 virus infection. Submitted for publication.

Supplementary figure 7

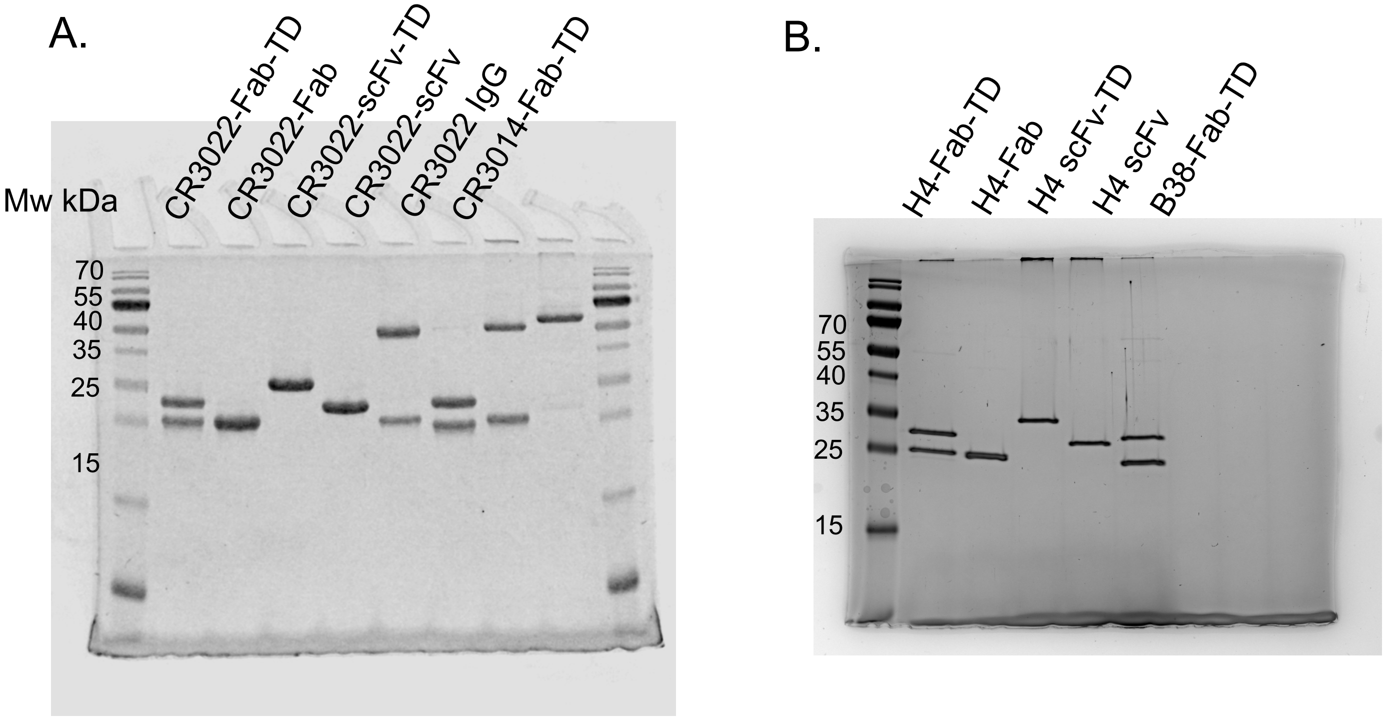

Supplementary figure 7: Uncropped polyacrylamide gels from figure 2A and B

Supplementary Figure 8

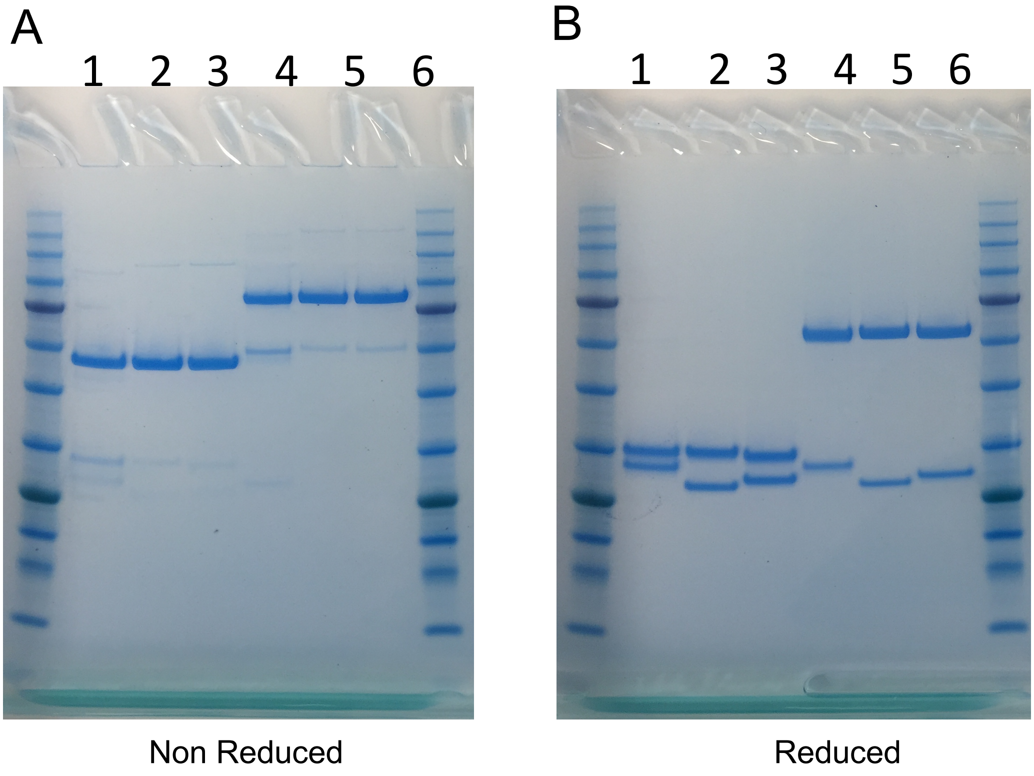

1. REGN10987 Fab-TD
2. REGN1033 Fab-TD
3. CB6 Fab-TD
4. REGN10987 mIg-TD
5. REGN10933 mIg-TD
6. CB6 mIg-TD

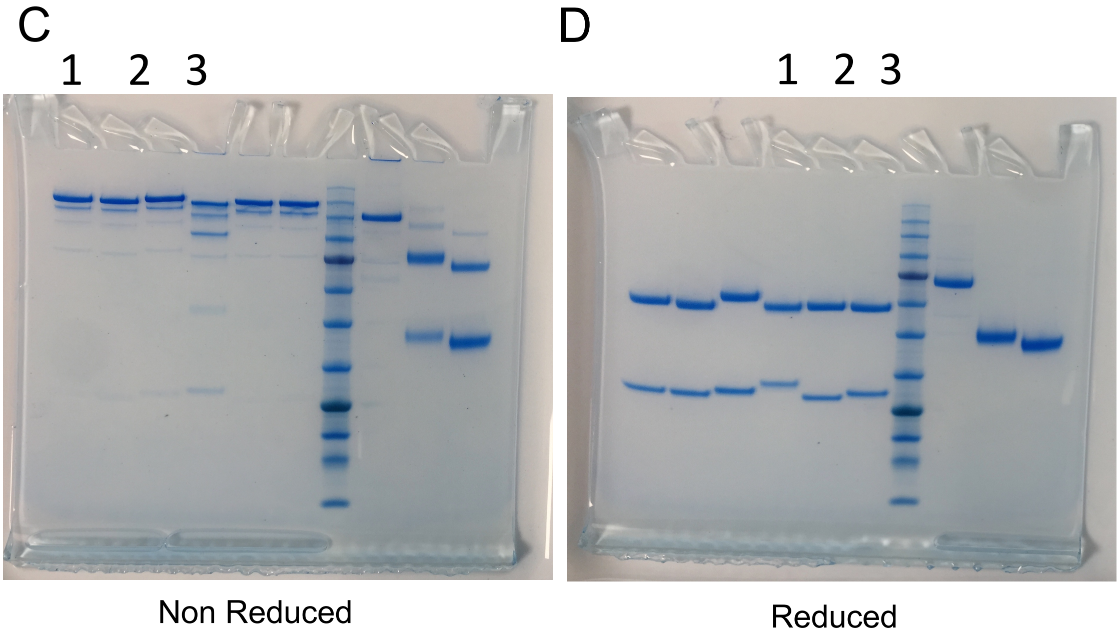

1. REGN10987 Ig-TD
2. REGN10933 Ig-TD
3. CB6 Ig-TD

Supplementary Figure 8: Uncropped PAGE analysis of reformatted clinical stage SARS-CoV-2 mAbs from Supplementary Figure 2

Gels C and D are the Ig-TD quads (indicated in lanes 1, 2 and 3 that were separated on SDS-PAGE alongside other non-relevant proteins in the unnumbered lanes.
